# Control of Western Corn Rootworm (*Diabrotica virgifera virgifera*) Reproduction through Plant-Mediated RNA Interference

**DOI:** 10.1038/s41598-017-12638-3

**Published:** 2017-10-03

**Authors:** Xiping Niu, Adane Kassa, Xu Hu, Jonathan Robeson, Mollie McMahon, Nina M. Richtman, Joseph P. Steimel, Bliss M. Kernodle, Virginia C. Crane, Gary Sandahl, Julie L. Ritland, James K. Presnail, Albert L. Lu, Gusui Wu

**Affiliations:** 1 DuPont Pioneer, 7300 NW 62nd Ave., Johnston, IA USA; 2 Evogene Ltd, Saint Louis, MO USA

## Abstract

RNA interference (RNAi) in transgenic maize has recently emerged as an alternative mode of action for western corn rootworm (*Diabrotica virgifera virgifera*) control which can be combined with protein-based rootworm control options for improved root protection and resistance management. Currently, transgenic RNAi-based control has focused on suppression of genes that when silenced lead to larval mortality. We investigated control of western corn rootworm reproduction through RNAi by targeting two reproductive genes, *dvvgr* and *dvbol*, with the goal of reducing insect fecundity as a new tool for pest management. The results demonstrated that exposure of adult beetles, as well as larvae to *dvvgr* or *dvbol* dsRNA in artificial diet, caused reduction of fecundity. Furthermore, western corn rootworm beetles that emerged from larval feeding on transgenic maize roots expressing *dvbol* dsRNA also showed significant fecundity reduction. This is the first report of reduction of insect reproductive fitness through plant-mediated RNAi, demonstrating the feasibility of reproductive RNAi as a management tool for western corn rootworm.

## Introduction

The western corn rootworm (WCR), *Diabrotica virgifera virgifera* (Coleoptera: Chrysomelidae), is one of the most economically important and invasive pests of maize in the United States and Europe^1,2^. Currently, WCR damage is managed with crop rotation, broad-spectrum soil insecticides^3^, and transgenic crops expressing crystalline (Cry) proteins from *Bacillus thuringiensis* (Bt)^2^. Insect resistance to transgenic traits continues to emerge as a threat to the long-term durability of Bt crops^4^. Therefore, new modes of action control will be important for sustainable and durable WCR management^5,6^ in the future.

RNA interference (RNAi) pathways have been found in many eukaryotes including insects^7^, and transgenic crops utilizing RNAi represent a promising new tool for insect pest control and management^8^. Some insect orders such as Coleoptera are sensitive to environmental RNAi (ingested double-stranded RNA (dsRNA)) and display strong RNAi responses^9,10^. This type of responses in WCR has allowed the development of transgenic maize plants using insecticidal (or lethal) RNAi that show root protection against rootworm^8,10–12^. Successful WCR RNAi targets used for these transgenic maize plants include *α-tubulin* gene^8^ *, V-ATPase* subunits A^8^ and C^12^ genes, an intracellular protein trafficking pathway gene *snf7* ^8,10,13^ and a midgut expressed gene *ssj1* ^11^. In addition, WCR females exposed to parental RNAi that suppress two embryonic developmental genes, *hunchback* and *brahma*, exhibited reduced egg production and hatch rate in diet assay^14^. We postulate that targeting genes specifically involved in insect reproduction may provide a new mode of action for WCR control.

Vitellogenin, the precursor of the major yolk protein in most oviparous animals, is transported into oocytes by the vitellogenin receptor (VgR) through an endocytic pathway^15^. The *vgr* gene is highly expressed in ovarian tissue in female insects, and VgR has been identified and studied in insects of several orders^16–18^. Reduced fecundity has been reported when *vgr* expression is suppressed by a mutation in silkworm (*Bombyx mori*)^19^ or by RNAi in brown planthopper (*Nilaparvata lugens*)^16^. Similarly, suppression of the *boule* (*bol*) gene in sawfly (*Athalia rosae*) affects meiosis during spermatogenesis leading to a reduction of sperm maturation divisions and a male sterile phenotype^20^. The *bol* gene was first described in the molecular genetic analysis of spermatogenesis mutants generated by a P-transposable element in Drosophila^21^. BOL is an RNA-binding protein with an RNA Recognition Motif (RRM) domain^22^. It shares homology with the DAZ (Deleted in Azoospermia) protein outside the RRM domain, and mutations in this protein cause severe sperm reduction in animals^23^. Based on this previous characterization of *vgr* and *bol* as essential reproductive genes in other insect species, we chose to evaluate WCR homologs of these genes, *dvvgr* and *dvbol*, as targets for reproductive RNAi in WCR. The impact to fecundity by suppressing gene expression was tested by feeding WCR larvae and adults artificial diet incorporating dsRNA derived from *vgr* or *bol* (ds*vgr* and ds*bol*). In addition, transgenic maize plants expressing ds*vgr* and ds*bol* were generated to determine whether fecundity would be affected by larvae feeding on transgenic roots to demonstrate the potential for WCR reproductive RNAi as a transgenic trait. To the best of our knowledge, this is the first report of plant-mediated reproductive RNAi for insect control, and it represents a new rootworm management approach.

## Results

### Identification and expression of WCR VgR and BOL

**Figure 1:**
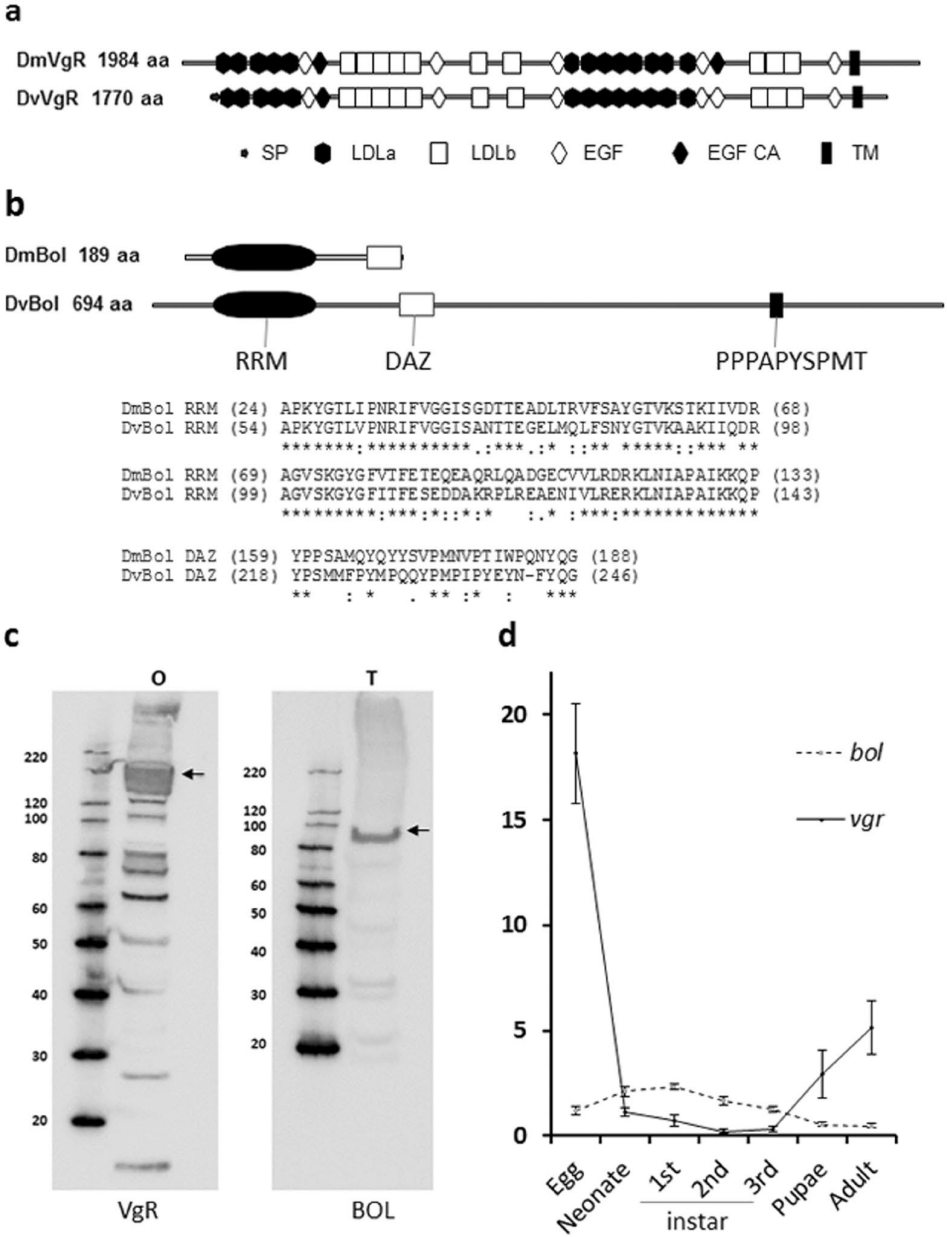
Predicted protein domains of *Diabrotica virgifera virgifera* VgR and BOL and expression. (**a**) VgR protein domain structure. *Drosophila* (DmVgR) and DvVgR protein structures are compared and the locations of the signal peptide (SP, black arrow), density lipoprotein receptor class A (LDLa, black hexagon), low-density lipoprotein receptor class B (LDLb, open rectangle), epidermal growth factor-like (EGF, open diamond), EGF-like with calcium binding site (EGF CA, black diamond), and transmembrane (black rectangle) domains are indicated. (**b**) BOL protein domain structure. DmBol and DvBol protein structures are compared and the locations of RNA recognition motif (RRM, black oval), Deleted in Azoospermia (DAZ, open rectangle), and PPPAPYSPMT regions are indicated. Sequence alignments of RRM and DAZ between DmBol and DvBol are shown with indications of identical amino acid (*), high similarity (:), and weak similarity (.) amino acids. The numbers in parenthesis show the beginning and ending positions of amino acids in the BOL proteins. (**c**) Western blot detection of VgR and BOL proteins in *D. virgifera virgifera* adult reproductive tissues. Proteins were extracted from the dissected ovary and testes using different methods, as described in the Supplementary method A. Loaded samples represent the equivalent of 1 ovary (O) or 2.5 testes (T), based on optimized conditions (Supplementary Fig. 2). VgR and BOL were detected using polyclonal peptide antibodies and protein size was estimated using a standard marker. Full-length blots are presented in the Supplementary Fig. 2c. (**d**) Relative mRNA expression of *D. virgifera virgifera* target genes in different life stages. qRT-PCR was used to examine gene expression of *vgr* and *bol* after feeding on diet incorporated with 50 ng μl^−1^ or different doses of *vgr* and *bol* fragment dsRNA. Relative expression analysis (mean ± SE) was based on *bol* and *vgr* expression in individual insects (n = 12) at each life stage, after being normalized to the expression of the reference gene, *dvrps10*.

We selected two WCR reproductive genes, *vgr* and *bol*, based on the following: [1] homology to reproductive genes listed in FlyBase^24^ or to RNAi lines showing sterile phenotype^25^; [2] previously reported to have reproductive functions; and [3] likely expressed in germ cells. A putative full-length cDNA sequence encoding WCR VgR was identified from the transcriptome assembled from WCR adult females by blastP search and compared to other insect VgRs, including *Drosophila* (DmVgR)^15,26^. VgR proteins belong to the low-density lipoprotein receptor (LDLR) family, which are membrane-bound proteins^15^. The *dvvgr* (or *vgr*) cDNA has an open reading frame of 5313 nucleotides (nt) which encodes a large protein of 1770 amino acids (aa) that is predicted to contain multiple domains typically found in other insect VgRs (Fig. 1a). These domains include a signal peptide, five low-density lipoprotein receptor class A (LDLa) repeats, two epidermal growth factor (EGF)-like repeats, five low-density lipoprotein receptor class B (LDLb) repeats, one EGF, two LDLb repeats, and one EGF region, followed by eight LDLa, two EGF, three low homology LDLb repeats, one EGF-like region and a transmembrane region at the C-terminus. Although the identity between DvVgR and DmVgR is only 28%, the overall protein architectures are very similar (Fig. 1a). Overall identity of DvVgR to other insect VgRs ranges from 47% in the Coleoptera order to 26% in the Lepidoptera order (Supplementary Fig. 1a).

DvBOL was identified from the transcriptome assembled from adult WCR testes by a blastP search using *Drosophila* DmBOL^22^. The *dvbol* (or *bol*) cDNA encodes a 694 aa protein with a conserved RRM domain found in DmBOL (Fig. 1b). While the identity of RRM domain between DvBOL and DmBOL is 72% (over 90 aa), the putative DAZ domain of DvBOL is 32% identical to that of DmBOL (over 31 aa). DvBOL is considerably larger than DmBOL protein (694 aa vs 189 aa), but DvBOL is similar in size to *Tribolium castaneum* TcBOL, which is another family in Coleoptera (694 aa vs 634 aa). There was no other region outside of the RRM domain that was conserved across the analyzed insect BOL proteins in Genbank, but one segment of amino acid sequence (PPPAPYSPMT) present in DvBOL is conserved in BOL proteins from multiple hymenopterans, including *Athalia rosae* ArBOL-2 isoform, *Atta colombica* and *Diachasma alloeum* (Fig. 1b). Overall, the identity of DvBOL to other insect BOLs ranges from 47% in the order Coleoptera to 9% in the order Lepidoptera (Supplementary Fig. 1b).

**Figure 2:**
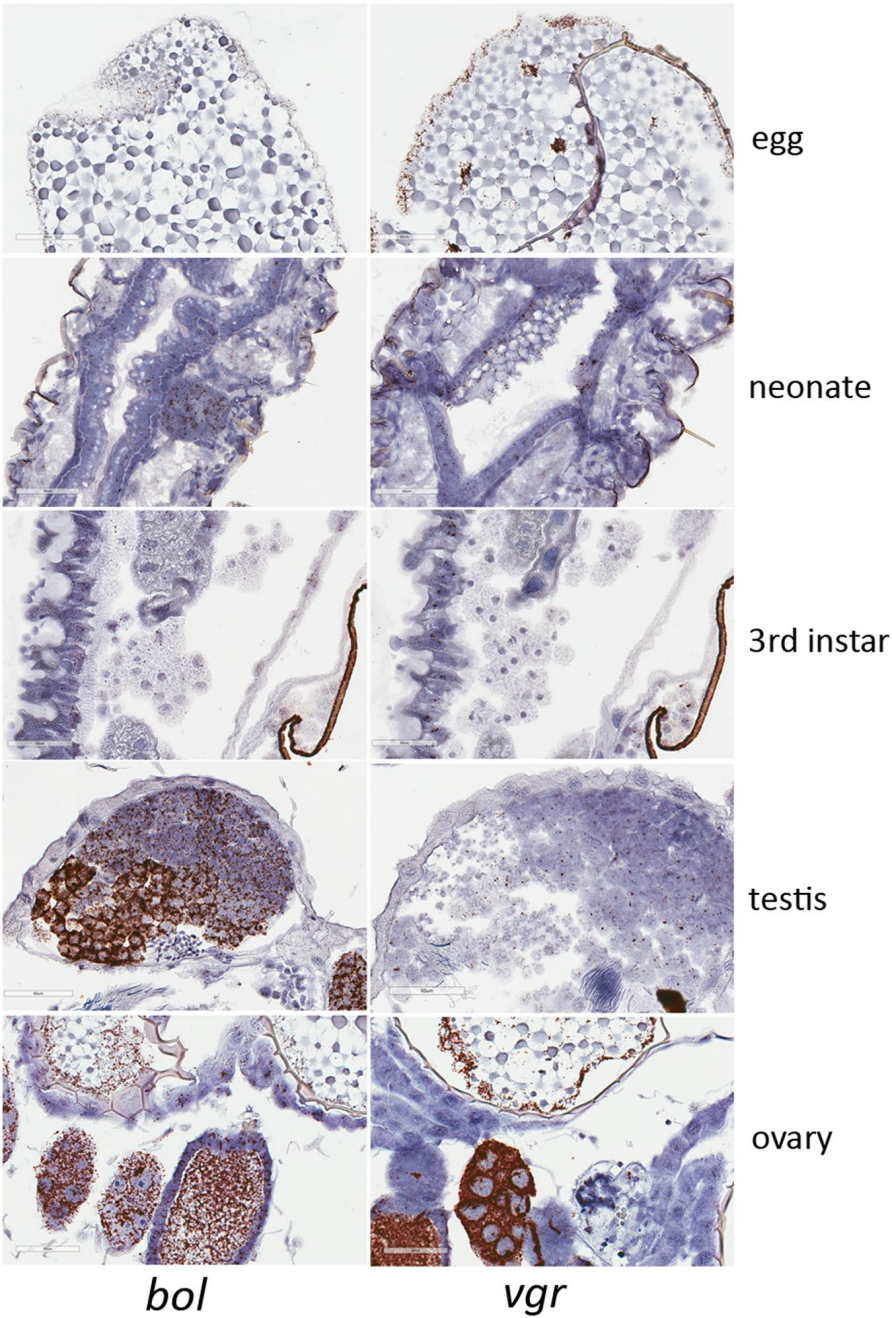
Visualization of *vgr* and *bol* mRNA expression during different life stages by *in situ* hybridization. Representative *Diabrotica virgifera virgifera* sample sections (Supplementary Fig. 3) were collected from the egg (1), neonate (2), 3^rd^ instar of larvae (3), and dissected testes (4) and ovaries (5) from adults. All samples were hybridized with the *vgr* and *bol* probes and an RNAscope® negative control probe (*Bacillus subtilis* dihydrodipicolinate reductase (*dapB*) gene) were included for 3^rd^ instars, as described in the Supplementary Fig. 3. Expression of *bol and vgr* mRNA are demonstrated in the different life stages and the reproductive tissues. Images were captured at 40x magnification with 60 µm scale bars.

VgR and BOL proteins were extracted from adult WCR reproductive tissues and analyzed by western blot. The VgR and BOL proteins were detected at the predicted MWs of 197.8 kDa (ovary) and 75.9 kDa (testes), respectively (Fig. 1c and Supplementary Fig. 2). Expression of bol and vgr mRNA was analyzed by real-time quantitative reverse transcription PCR (qRT-PCR) using individual whole insects representing different life stages (Fig. 1d), and by *in situ* hybridization (ISH) methods on specific life stages as well as dissected reproductive tissues from the WCR adults (Fig. 2, Supplementary Fig. 3). The mRNA expression of *vgr* showed clear differences depending on the life stage. For example, *vgr* mRNA expression was about 10-fold higher in the egg and between 2–4 fold higher in pupae and adults than in larvae. On the other hand, expression of *bol* mRNA was similar across life stages (within 2 fold) by qRT-PCR and it was highly expressed in testis and moderately expressed in the ovary compared to *vgr* (Fig. 2).

### Fecundity of WCR adults exposed to ds*vgr* or ds*bol*

**Figure 3:**
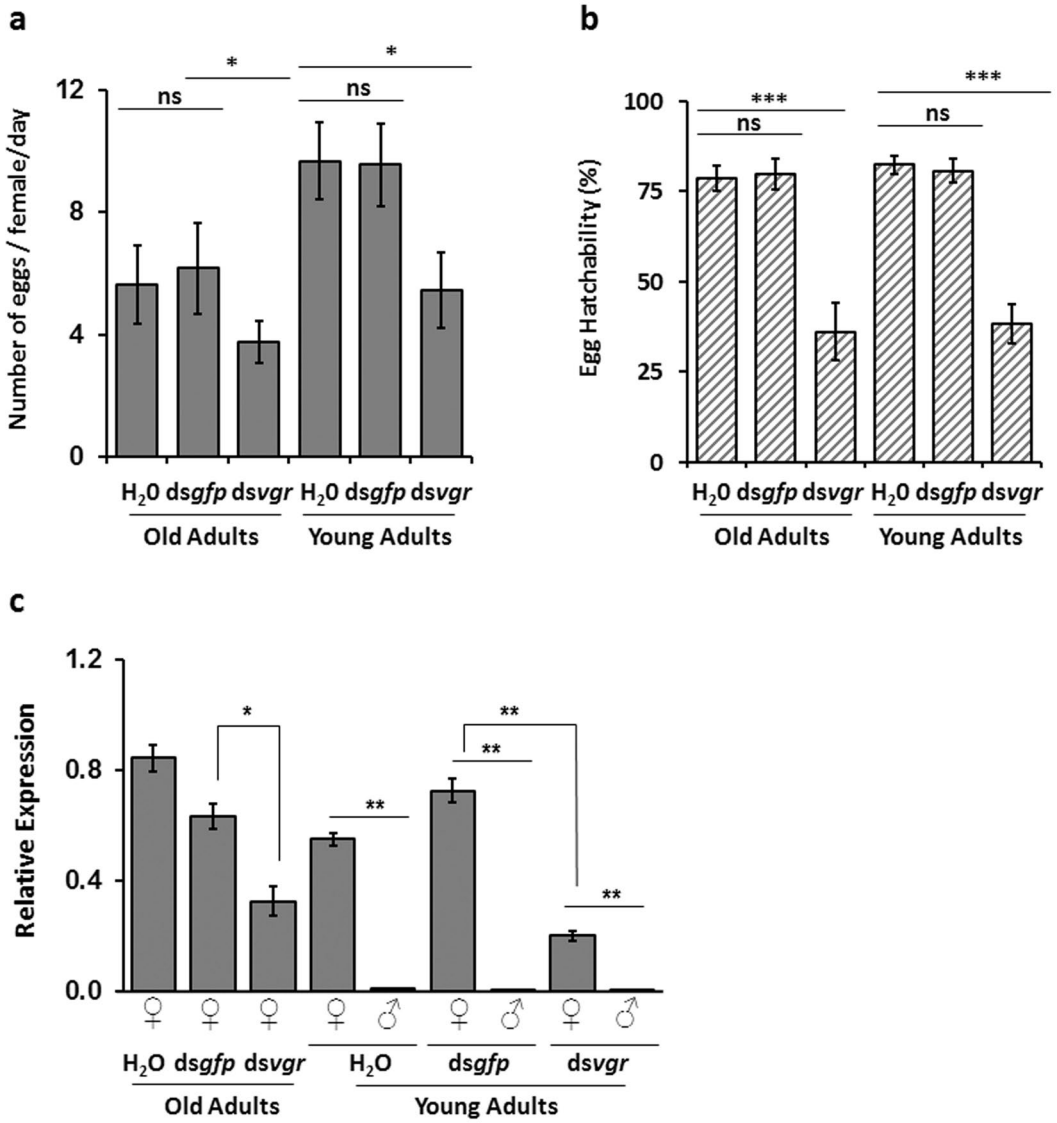
Fecundity and mRNA expression of adult *Diabrotica virgifera virgifera* exposed to dsRNA *vgr* (ds*vgr*). For young adults (<5 days old; 50 pairs) and for old adults (>11 days old; 50 mated females) were exposed individually to sterile deionized water (control), 100 ng μl^−1^ *gfp* dsRNA (ds*gfp*), or 100 ng μl^−1^ *vgr* dsRNA (ds*vgr*) in artificial diet for 24 h. Exposed beetles were used to assess fecundity. Least square means pairwise comparison *P*-values: >0.05 (ns; not significant), <0.05*, <0.01**, <0.0001***. (**a**) Number of eggs laid per female per day (mean ± SE; n = 13 to 14). Treatment main effect *P* = 0.002; Age main effect *P* = 0.0028; Treatment *age interaction *P* > 0.05. (**b**) Percent egg hatchability by treatment and age group (mean ± SE; n = 13 −14; 12–226 eggs/day). Treatment main effect *P* < 0.001; Age main effect *P* > 0.05; Treatment *age interaction *P* > 0.05. (**c**) Gene suppression analysis of WCR old adults 4 days after dsRNA treatment and young adults 8 days after dsRNA treatment (mean ± SE; n = 4). Relative expression of *vgr* mRNA by qRT-PCR assay is shown for each treatment using *rps10* as a reference and normalized to *vgr* expression in water control.

The genes of *vgr* and *bol* from WCR were evaluated for the impact of suppression on adult beetles in diet feeding assays. Since *vgr* is highly expressed in the ovary, it is possible that the age of the adult females may influence the effect of dsRNA treatments. Thus, experiments testing the impact of RNAi of *vgr* gene on fecundity were performed using young (<5 days old) and old adults (>11 days old) (50 female and male pairs of young adults and 50 mated female old adults) exposed to dsRNA in artificial diet (containing water control, 100 ng µl^−1^ of ds*gfp*, or ds*vgr*) for 24 h. Two parameters for fecundity (egg production and egg hatch rate) were assessed for each treatment. These parameters were used to calculate the net reduction in fecundity (NRF). Exposure of young and old adults to ds*vgr* led to a significant reduction in egg number, egg hatch rate, and *vgr* expression compared to water and *gfp* controls (Fig. 3a–c). The overall NRF following dietary exposure to ds*vgr* was 50.8 ± 20.3% and 75.9 ± 4.4% for old and young adults, respectively. Under similar conditions, exposure of old adults to 100 ng μl^−1^ dsRNA *bol* (ds*bol*), showed no significant change in egg production and egg hatch rate, despite detectable gene suppression (Supplementary Fig. 4). Further dose-response analysis was performed by exposing old adults to increasing concentrations of ds*vgr* (0.01, 0.1, 1, 10, and 75 ng μl^−1^) and monitoring fecundity endpoints. Old adults exposed to ds*vgr* concentrations ranging from 0.1 to 10 ng μl^−1^ resulted in 46.5 ± 12.5% to 75.4 ± 11.3% lower fecundity, indicating that lower doses were still effective (Supplementary Table 1).

### Effect on adult WCR fecundity by larval exposure to ds*vgr* or ds*bol*

**Figure 4:**
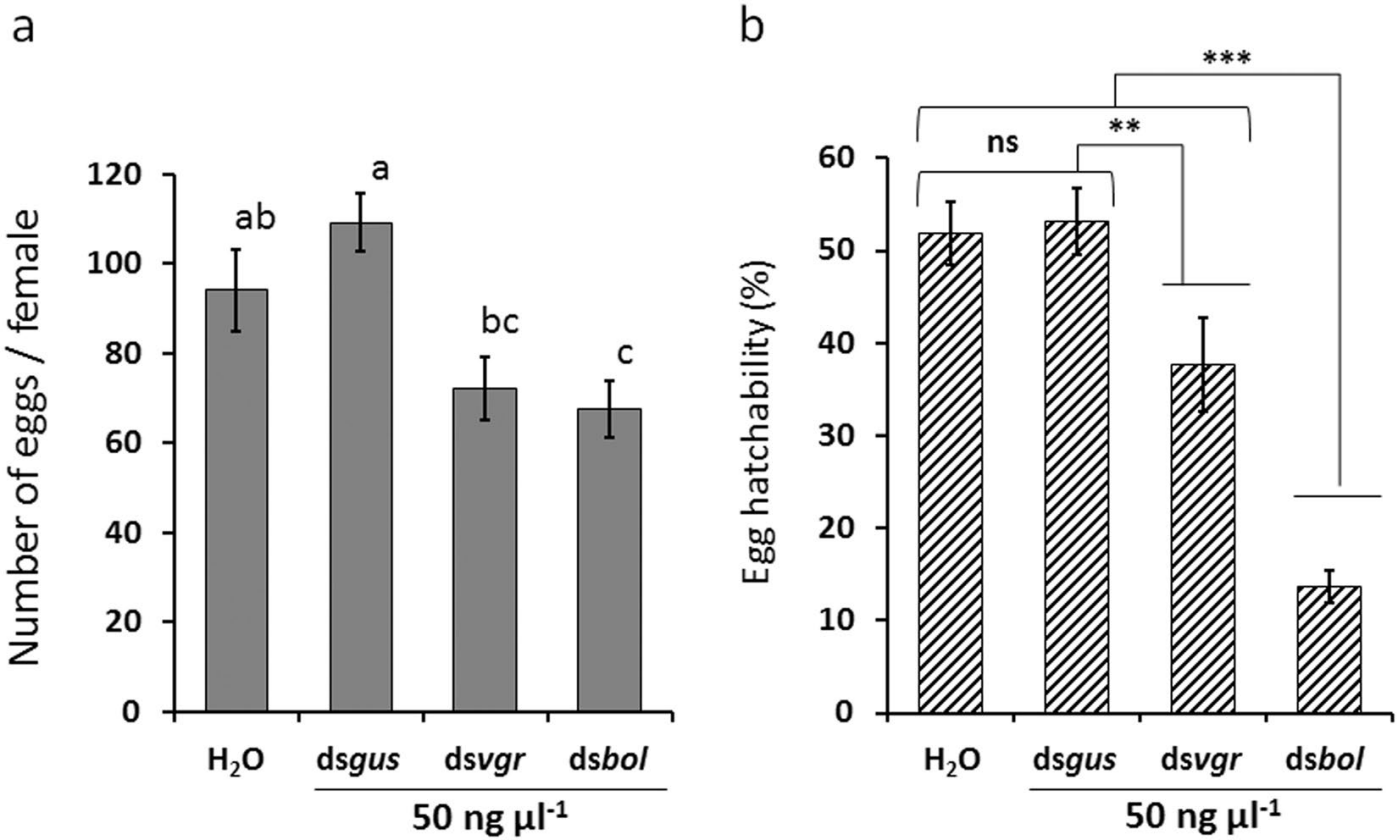
Fecundity of *Diabrotica virgifera virgifera* exposed to ds*vgr* and ds*bol* at 3^rd^ instar stage. Fecundity was assessed in adult corn rootworm that were exposed for 24 h to 50 ng μl^−1^ ds*gus*, ds*vgr*, ds*bol* or water (control) as 3^rd^ instar larvae. Least square means pairwise comparison *P*-values: >0.05 (ns; not significant), <0.01**, and <0.001***. (**a**) Egg production per female per 5-day (mean ± SE; n = 9). Treatment main effect: df = (3, 16); *F* = 5.28; *P* = 0.01). Bars followed by the same letters are not significantly different (**b**) Percent egg hatchability (mean ± SE; n = 9; and 95 to 174 eggs per observation). Treatment main effect: df = (3, 16); *F* = 26.3; *P* < 0.0001).

**Figure 5:**
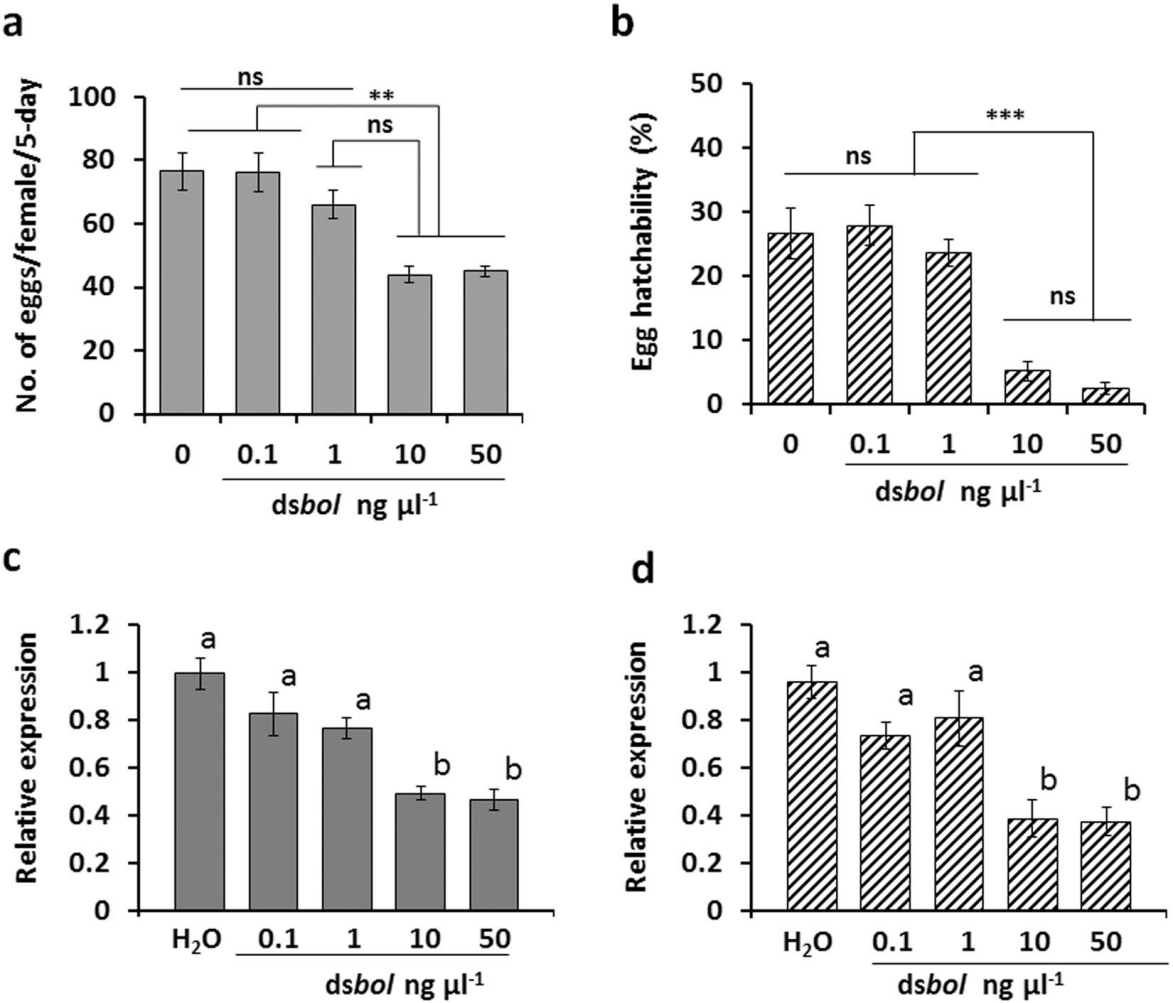
Fecundity and mRNA expression analyses of *Diabrotica virgifera virgifera* exposed to different concentrations of ds*bol* at 3^rd^ instar stage. Fecundity and mRNA expression were assessed in adult *Diabrotica virgifera virgifera* that were exposed to different concentrations (0.1, 1, 10, and 50 ng µl^−1^) of ds*bol* in diet as 3^rd^ instar larvae. Least square means pairwise comparison, *P*-values: >0.05 (ns; not significant), <0.01**, <0.0001***. (**a**) Number of eggs produced per female per 5-day (mean ± SE; n = 20). Dose main effect: df = (4, 51); *F* = 3.84; *P* = 0.0084). (**b**) Percent egg hatchability (mean ± SE; n = 20; 38 to140 eggs per observation for total about 1800 to 2008 eggs per treatment). Dose main effect: df = (4, 60)_;_ *F* = 16.2; *P* < 0.0001). (**c,d**) Relative mRNA expression (mean ± SE; n = 6) of *bol* mRNA in 3rd instar larvae (left panel) and pre-oviposition adults (right panel) was assessed by qRT-PCR. Bars followed by the same letters are not significantly different. Relative expression is shown for each treatment using *rps10* as a reference and after normalizing to *bol* expression in the control.

We also investigated if larval exposure to ds*vgr* or ds*bol* would cause a similar effect on fecundity as observed by adult exposure. We exposed 11-day-old 3^rd^ instar larvae to WCR larval diet mixed with 50 ng µl^−1^ of ds*bol*, ds*vgr*, ds*gus* (control) or sterile water. Relative mRNA expression of *vgr* and *bol* from larvae were measured by qRT-PCR and ISH. Exposed WCR larvae were allowed to complete life-cycle development and emerged adult beetles were collected, sexed and used for subsequent fecundity studies (egg production and egg hatch rate). Oral ingestion of ds*bol* and ds*vgr* at the 3^rd^ instar larval stage resulted in a significant reduction in adult beetle egg production and hatch rate compared to ds*gus* and water controls (Fig. 4). The estimated NRF relative to water control was 80.9% (±2.7) and 42.3% (±10.2) for ds*bol* and ds*vgr*, respectively. Subsequent dose-response experiment performed on 3^rd^ instar using a range of ds*bol* concentrations (0.1, 1, 10, and 50 ng µl^−1^) showed that 10 ng µl^−1^ was a high enough concentration to cause a significant reduction in emerged adult egg production, egg hatchability (Fig. 5a,b) and NRF (87.8 ± 4.8%). The 10 ng µl^−1^ was also high enough to reduce mRNA expression in both 3^rd^ instars (Fig. 5c) and pre-oviposition adults (Fig. 5d). In contrast, ds*vgr* reduced fecundity by only 12.5 ± 14.2% at 10 ng µl^−1^ (Supplementary Table 2). Target-specific knockdown of *bol* and *vgr* was confirmed in 3^rd^ instar larva collected 2 days after 50 ng µl^−1^ dsRNA exposure (Supplementary Fig. 5a,b) and this suppression was maintained in pre-oviposition adults for at least 25 days after beetle emergence (Supplementary Fig. 5c,d). No detectable morphological changes were observed in dissected reproductive tissues (Supplementary Fig. 6) even though suppression of *bol* mRNA was detected by ISH in the testes of ds*bol* treated samples compared to ds*gus* treated samples (Supplementary Figs 7 and 8).

### Fecundity of WCR feeding on transgenic plants expressing ds*vgr* or ds*bol*

**Figure 6:**
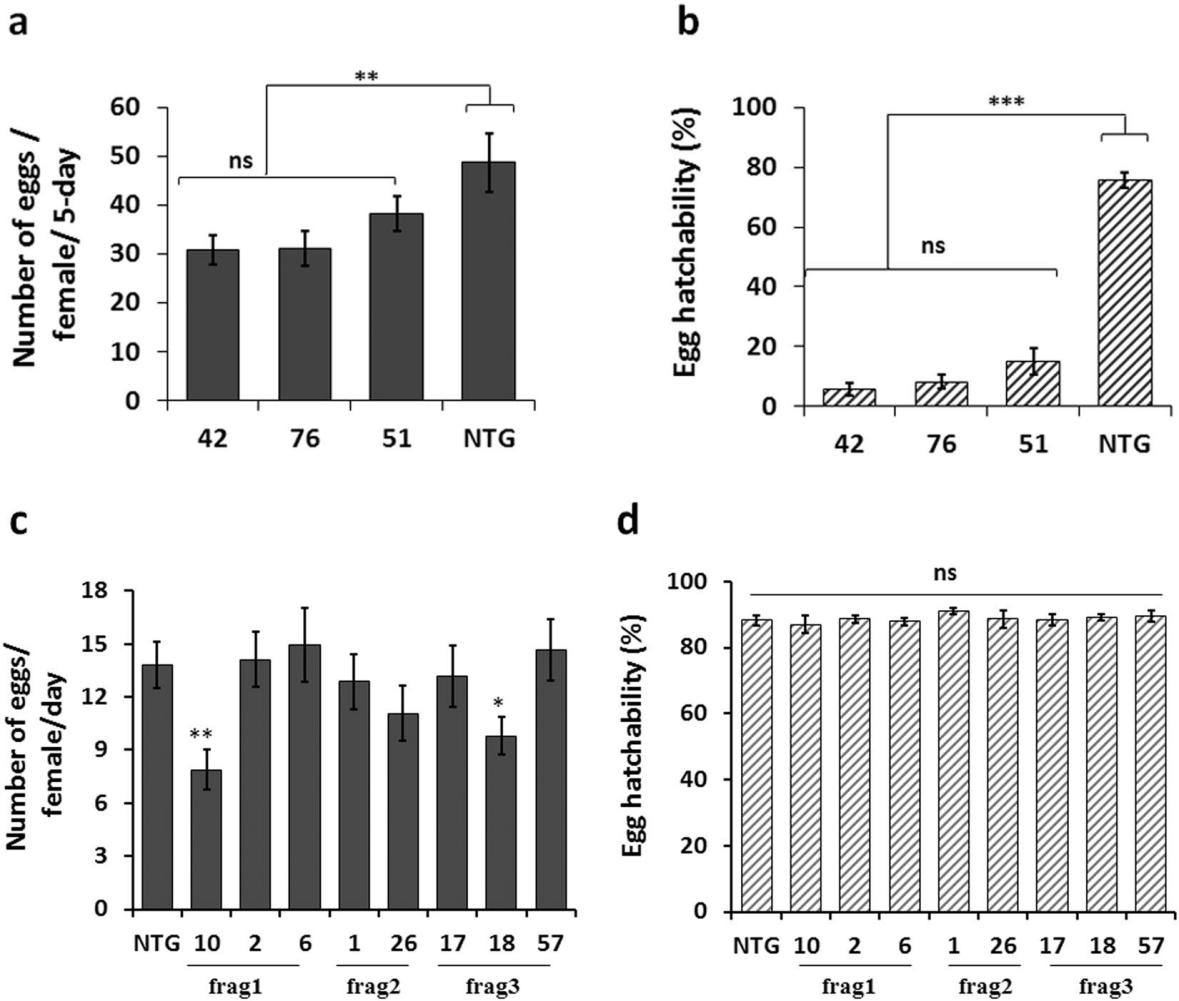
Fecundity of *Diabrotica virgifera virgifera* feeding on plants expressing *vgr and bol* dsRNA transcripts. Fecundity was assessed in adult corn rootworm that fed transgenic maize lines expressing the *bol* or *vgr* dsRNA transcript during the larval development period. Treatments included three *bol* transgenic lines (42, 76, and 51) and eight *vgr* transgenic lines from three transgenic constructs [(frag 1: Line 10, 2, 6); (frag 2: Line 1, 26) and (frag 3: Line 17, 18, 57)] and a non-transgenic control (NTG). Least square means pairwise comparison, *P*-values: >0.05 (ns; not significant), <0.01**, <0.0001***. (**a**) Egg production per female per 5-day (mean ± SE; n = 15). Event main effect: df = (3, 32); *F* = 3.71; *P* < 0.05, time main effect: df = (3, 32); *F* = 6.35; *P* < 0.001, event*time interaction: df = (12, 32); *F = *5.45; *P* = < 0.0001. (**b**) Percent egg hatchability (mean ± SE; n = 15; and 51 – 112 eggs per observation). Event main effect: df = (3, 32); *F* = 36.1; *P* = < 0.001, time main effect: df = (4, 32); *F* = 9.76; *P* < 0.001, event*time interaction: df = (12, 32); *F* = 0.76; *P* = > 0.05. (**c**) Egg production per female per day. (mean ± SE; n = 9). Event main effect: df = (8, 136); *F* = 2.15; *P* = 0.0349, Day main effect: df = (8, 136); *F* = 2.15; *P* < 0.001, event*day interaction: df = (64, 136); *F* = 1.05, *P* > 0.05. (**d**) Percent egg hatchability (mean ± SE; n = 6; and 103 – 224 eggs per observation). Event main effect: df = (8, 82); *F* = 0.46; *P* > 0.05, Day main effect: df = (8, 82); *F* = 9.91; *P* < 0.001, event*day interaction: df = (40, 82); *F* = 1.12; *P* > 0.05.

To test whether transgenic maize plants expressing *vgr* and *bol* dsRNA transcripts (Supplementary Fig. 9a) could result in a reduction of WCR fecundity, four-week-old maize T1 transgenic seedlings were infested with WCR eggs in the greenhouse. Beetles emerged from the infested transgenic plants were collected and maintained in the laboratory to assess fecundity. Exposure of neonates to transgenic plants expressing ds*vgr* or ds*bol* transcripts had no significant effect on the adult emergence compared with non-transgenic plants (Supplementary Fig. 10a and b). However, WCR larvae feeding on the three transgenic plant lines expressing the ds*bol* transcript resulted in a significant reduction in egg production and egg hatch rate of the emerged adult beetles (Fig. 6a and b), which resulted in an overall NRF ranged from 84.1 ± 5.8 to 95.3 ± 2.2%. This effect was mainly due to a significant reduction in egg hatch rate (80.8 ± 5.6–92.7 ± 2.7%) when compared to non-transgenic control (Fig. 6b). WCR larvae feeding on eight transgenic plant lines expressing the ds*vgr* transcripts (three different fragments) showed less impact on fecundity as there were only two lines where emerged adults showed a significant reduction in egg production (Fig. 6c) but none of them influenced egg hatch rate (Fig. 6d). Molecular analyses confirmed that both long dsRNA transcripts and siRNA were expressed in the transgenic lines (Supplementary Fig. 9b and Supplementary Table 3).

### Male-specific effect of ds*bol* exposure shown by reciprocal crossing

**Figure 7:**
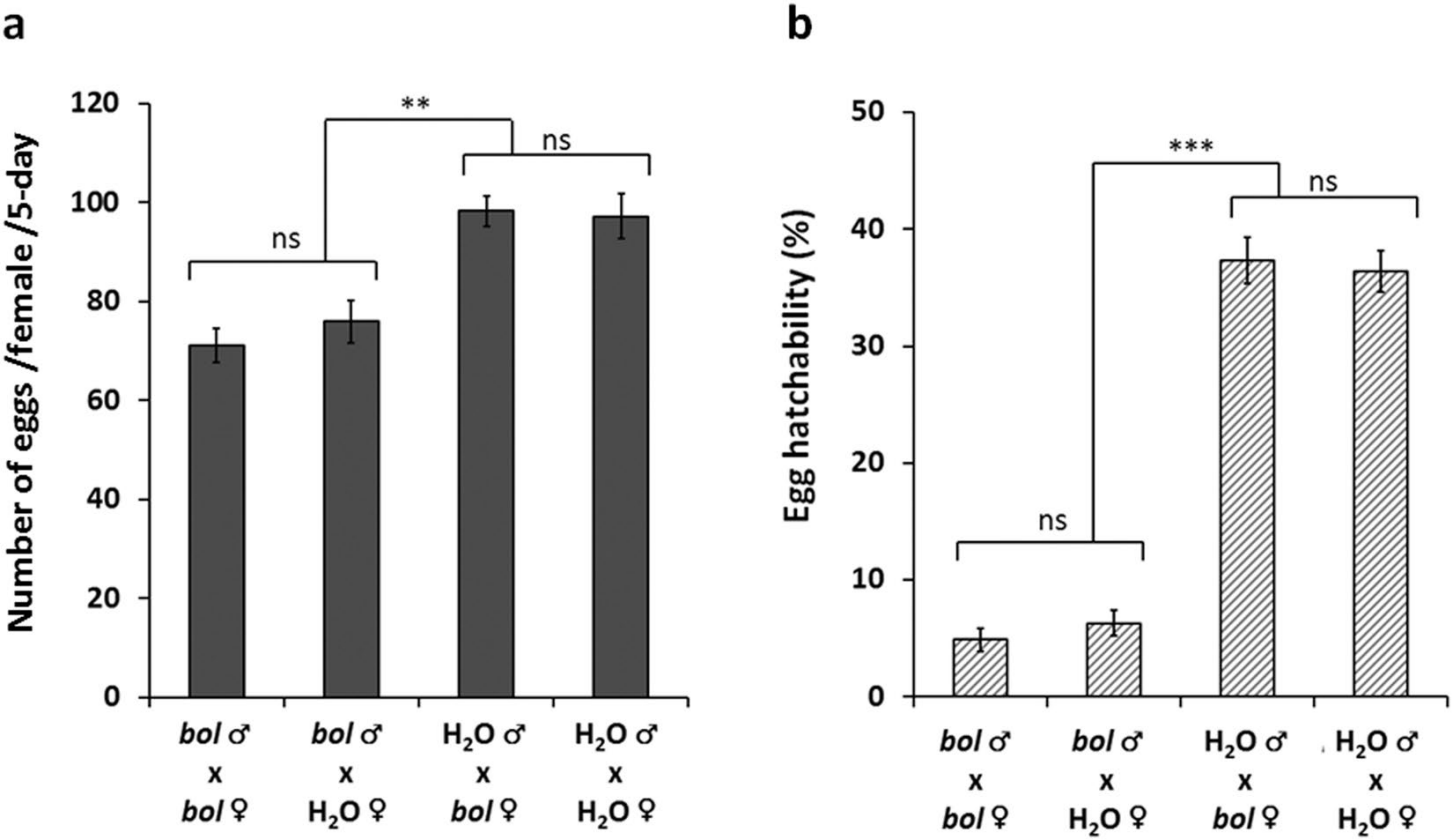
Sex-specific effects of ds*bol* exposure at 3^rd^ instar stage via reciprocal crossing. Fecundity was assessed in adults that were exposed to 50 ng μl^−1^ of ds*bol* or water control as 3^rd^ instar larvae. Four reciprocal crossing combinations (*bol* ♂ x *bol* ♀; bol ♂ x H_2_O ♀; H2O ♂ x *bol* ♀; H2O ♂ x H_2_O ♀) were assessed. Least square means pairwise comparison *P*-values: >0.05 ns (not significant), <0.01**, <0.001***. (**a**) Egg production per female per 5 -day (mean ± SE; n = 30) and (**b**) Percent egg hatchability (mean ± SE; n = 30; 83 to 151 eggs per observation). For both egg number and hatchability data, crossing and time main effects were significant (*P* < 0.01) and crossing*time interaction was not significant (*P* > 0.05).

The *bol* gene was discovered to impact male spermatogenesis in Drosophila^21^, as well as other insects^20,27^. To further characterize *dvbol*, a reciprocal crossing experiment was conducted to assess the effect on fecundity if only one of the adults (either the male or female) in the mating pair was exposed to ds*bol*. Eleven-day-old 3^rd^ instar larvae (n = 3780) were exposed to 50 ng µl^−1^ ds*bol* or water (control) for one day and allowed to complete their development and to emerge as adults. Four reciprocal crossing combinations were assessed: exposed ds*bol* males mated with exposed ds*bol* females (*bol* ♂ x *bol* ♀); exposed ds*bol* males mated with unexposed females (*bol* ♂ x H_2_O ♀); unexposed male mated with exposed ds*bol* female (H_2_O ♂ x *bol* ♀), and unexposed males mated with unexposed females (H_2_O ♂ x H_2_O ♀). The mating of exposed ds*bol* males with unexposed females (*bol* ♂ x H_2_O ♀) significantly reduced both egg number and egg hatch (Fig. 7), while the mating of unexposed males to exposed ds*bol* females (H_2_O ♂ x *bol* ♀) had no significant effect on fecundity (Fig. 7). The results of the reciprocal crossing experiments and the observed suppression of *bol* mRNA in testes (Supplementary Fig. 7a) together support *dvbol’s* male-specific role in WCR reproduction.

## Discussion

Protection against rootworm in transgenic plants has been demonstrated using insecticidal RNAi targets^8,10–12^. We postulated that targeting reproductive genes using RNAi (reproductive RNAi) would be another approach using transgenic maize plants for WCR control and management. RNAi of reproductive genes has shown great promise for pest management with mosquitoes (*Aedes aegypti*)^27^ and with oriental fruit fly (*Bactrocera dorsalis*)^28^. These studies generated sterile males by feeding or injecting dsRNA against essential testis genes into the insect which resulted in reduced fecundity^20,27,28^. We in this study have shown that reproductive RNAi can be an effective approach for controlling WCR reproduction. Reduction of fecundity was observed when RNAi exposure occurred to WCR adult beetles as well as 3^rd^ instar larvae. More significantly, this effect on fecundity reduction was extended to WCR exposed to transgenic maize plants. We selected *bol* and *vgr* genes because they have been extensively characterized in their role in reproduction across multiple insect species. We have further characterized their tissue expression across developmental life stages and observed an expression profile (timing and tissue) consistent with their expected reproductive function. Furthermore, silencing of *dvvgr* and *dvbol* in WCR did not cause growth inhibition or mortality (adult beetle emergence) in diet- or transgenic plant-based assays. This allowed us to assess the effect of gene suppression directly on WCR fecundity. The observed fecundity reduction of emerged beetles that had fed on the roots of transgenic plants demonstrates the feasibility of WCR control using reproductive RNAi.

Expression of *dvbol* and *dvvgr* showed different patterns in larvae and suppression of these genes had differential effects on fecundity between larval and adult stages of WCR. The fecundity reduction of ds*vgr* was high with exposure to adult females but low with 3^rd^ instar larvae. In contrast, ds*bol*’s impact on fecundity was only observed by larval exposure (artificial diet and transgenic root). It has been reported that rootworm RNAi is more effective at larval stage than adult stage^29^. There are several possible explanations for the differences in fecundity that were measured from silencing of the two targets. One explanation may be differences of the sex-specific and age-dependent functions of the genes. WCR *vgr* is a female-specific gene that is highly expressed in ovaries which are fully developed in adult female beetles (post-emergence from pupae). In this study, *vgr* mRNA expression was found to be low at multiple larval stages but high in eggs and adults as measured by both qRT-PCR and ISH. As a result, ds*vgr* exposure to adult females would coincide with VgR’s function in transport of nutrients (including lipids and carbohydrates) to growing embryos within oocytes^15^, disruption of which would have a significant negative impact on fecundity. No effect on fecundity due to ds*vgr* larval exposure would be expected during developmental stages when *vgr* expression is low and VgR is not contributing to egg development. These results also suggest that ds*vgr* either does not persist from exposure at 3^rd^ instar larvae to adults or the dose accumulated during this time is not sufficient to significantly suppress the high levels of *vgr* expression in adults. In comparison, *bol* expression was observed to be relatively constant throughout all WCR life stages and was also detected in germline cells^20,30^ (Supplementary Fig. 3c). In the planarian *Schmidtea mediterranean*, two *bol* paralogs have been identified; *Smed-bol1* is required for meiotic progression, and *Smed-bol2* is required for the maintenance of the earliest male germ cells^31^. In higher animals (invertebrates and primates), ancestral *bol* was duplicated during evolution and 2–3 BOL family members (BOL, DAZ, and DAZL) have been shown to have more specified roles during germ cell development^23,32^. However, only one *bol* gene has been identified in the genome of WCR and Tribolium. It is possible that different transcript isoforms of a single invertebrate *bol* may have different functions throughout male germ cell development. Suppression of *bol* in WCR larvae is likely to interfere with one or more germline cell functions, including maintenance, renewing and pre-meiotic division^21^, leading to fecundity reduction in WCR adults. The reciprocal cross experiment of *bol* dsRNA confirms that WCR *bol* has a male-specific effect (Fig. 7), even though *bol* mRNA is expressed in the ovary (Fig. 2).

A second explanation for the observed differences in WCR response may be the difference of ds*vgr* and ds*bol* accumulation in transgenic maize plants. An impact on adult fecundity was observed because of exposure of 3^rd^ instar larvae to *bol* and *vgr* dsRNA using artificial diet bioassays. The dose administered in the diet bioassays was significantly higher than the expression levels found in transgenic plants, in part due to endogenous plant RNAi dicer activities^33^. The dsRNA accumulation in transgenic roots was in the range of 0.6 to 11 pg mg^−1^ fresh weight (Supplementary Table 3). This level was near the dsRNA LC_50_ threshold needed to elicit RNAi responses in Coleoptera^10^. However, it is unclear but conceivable that continuous exposure to a low dose of dsRNA over the full larval development period, as it is the case for larvae feeding on transgenic roots, can compensate for a single high dose exposure at 3^rd^ instar as in the diet assay. In the case of *dvbol*, a single diet exposure to 3^rd^ instar larvae of 10 to 50 ng µl ^−1^ ds*bol* produced comparable effects to WCR larvae exposed to roots of transgenic plants expressing *dvbol* dsRNA, from egg hatch to pupation. In contrast, continuous larval exposure to ds*vgr* from transgenic roots was either not sufficient to maintain suppression of *dvvgr* into adults or because *dvvgr* is highly expressed in ovaries, the amount of ds*vgr* expressed in roots was inadequate to reduce *dvvgr* expression in adults below the threshold needed to exert a significant effect on fecundity. The absence of an RNA-dependent RNA polymerase (RdRp) in insects^34^ implies that a RdRp-dependent amplification mechanism to spread silencing is lacking in insects. Consequently, cumulative dose and timing of exposure relative to target gene expression in the host organism is an important factor for successful suppression of reproductive genes both in an artificial diet system and through plant-mediated RNAi. These results suggest that although both *dvvgr* and *dvbol* are good targets for reproductive RNAi, *dvvgr* is more effective at the adult stage and *dvbol* is more effective during the larval stage. In a transgenic plant approach, this would suggest that ds*vgr* would be most effective if expressed in aerial tissues (pollen, silk, leaf) where adult WCR preferentially feed, while ds*bol* would be most effective when expressed in root tissue for larval exposure. Targeting suppression of both *dvvgr* and *dvbol* may be an interesting approach to maximize fecundity reduction since it would impact both males and females.

The potential of using RNAi-based transgenic plants to suppress WCR reproduction provides a useful alternative approach for rootworm control that is complementary to current insect control strategies. Reproductive interference has been exploited previously as a tool for insect control^35^ including tactics for manipulating pheromones, irradiation, pathogens, symbionts and genetic techniques^36^. The production of male sterile insects through sterile insect technique (SIT) by RNAi has also been proposed as a method of control^36^ particularly through the silencing of testis-expressed genes. WCR reproductive RNAi mediated through the transgenic delivery of dsRNA is not a stand-alone option for rootworm control, given that root protection is not expected from RNAi targeting these two reproductive genes. However, when combined with other pest management tools (and insecticidal traits) and given sufficient time, it may generate a significant benefit by suppressing pest populations, increasing the effectiveness of insect resistance management and improving root protection. For example, WCR reproductive RNAi pyramided with a WCR insecticidal active would lower the reproductive fitness of any adults that survive from larvae feeding on roots making it less likely to contribute to the development of resistance against the insecticidal active expressed in the transgenic plant. The release of sterile pink bollworm (*Pectinophora gossypiella*) in combination with Bt cotton significantly reduced the population of pink bollworm to the extent that insecticide sprays were no longer required^37^. Although this is an extreme example, it does show the effectiveness of population suppression through reproductive control as a component for integrated rootworm management.

In conclusion, *dvvgr* and *dvbol* are suitable gene targets for WCR reproductive RNAi. Double-stranded RNA targeting *bol* expressed in transgenic maize plants can down-regulate gene expression in WCR and effectively reduce the fecundity of WCR adult beetles. Transgenic plant mediated-reproductive RNAi offers a new tool for pest management which can be used with insecticidal traits to potentially enhance trait durability and efficacy.

## Methods

### Transcriptome assembly and protein analyses

The *D. virgifera virgifera* transcriptome was assembled as previously described^38^. Briefly, cDNA prepared from eggs, neonates, midguts of third instar larvae, testes from male and ovaries from female adults were sequenced by Illumina paired-end and 454 Titanium sequencing technologies. *De novo* transcriptome assemblies were performed using the Trinity method and the pooled assembly resulted in 81,277 contigs. The transcripts of *dvvgr* and *dvbol* were identified from transcriptome data set described above by blastP search using insect VgRs and BOL^15,22,26^. WCR VgR and BOL protein domains were predicted by InterProScan^39^. VgR and BOL proteins were extracted from dissected female and male corn rootworm reproductive tissues (Supplemental Methods A), and western blot analysis was used to detect the VgR and BOL protein in extracted WCR female and male reproductive tissues, respectively (Supplementary Fig. 2).

### Production of double-stranded RNA by *in vitro* transcription

DNA fragments of 155 to 250 base pair regions of *vgr* and *bol* cDNA sequences (Supplementary Table 4) were amplified from gBlock using Taq DNA polymerase (Integrated DNA Technologies, Inc. Coralville, Iowa) with a pair of gene specific primers or produced by overlapping extension by PCR using four complementary DNA oligodeoxyribonucleotide (oligo) primers^40^. The gene-specific primers also contained promoter sites for T7 RNA polymerase at the 5′ end of each primer or at external oligos for overlapping extension (Supplementary Table 5). The PCR product served as the template for dsRNA synthesis by *in vitro* transcription (IVT) using an MEGAscript kit (Life Technologies, Carlsbad, CA).

### Plant expression vectors and transformation

To demonstrate rootworm efficacy *in planta*, *dvvgr* and *dvbol* gene fragments were assembled into a suppression cassette designed to express dsRNA targeting the *vgr* or *bol* gene. The silencing cassette consisted of two 155 base pair stretches of *bol* (or *vgr*) and an intervening truncated maize ADH intron1 designed to support assembly into a dsRNA (Supplementary Fig. 9a). The constructs of *bol* and *vgr* were transformed via *Agrobacterium tumefaciens* into a commercial maize elite-inbred line, PHR03^41^. T0 maize transformants were transferred to soil and backcrossed with a PHR03 inbred line to generate T1 progeny.

### Quantitative Reverse Transcription PCR (qRT-PCR) and *In situ* hybridization (ISH)

The expression of *vgr* and *bol* gene was quantified from WCR eggs, neonates, 1^st^, 2^nd^, 3^rd^ instar, pupae, and adults after feeding on diet incorporated with 50 ng μl^−1^ or different doses of *vgr* and *bol* fragment dsRNA. The designs of primers and probe regions are listed in Supplementary Table 6. Gene expression was analyzed using one-step real-time qRT-PCR. The assay was run, with 3 replicates per sample, using a single-plex set up with Bioline Sensifast Probe Lo Rox kit (Taunton, MA) and analyzed using the 2^−ΔΔCt^ method based on the relative expression of the target gene and reference gene *dvrps10*. For *in situ* hybridization (ISH) analyses, target probes were designed by Advanced Cell Diagnostics (Hayward, CA) (listed in Supplementary Table 6). Insect samples were fixed in 10% neutral buffered formalin (4% formaldehyde) for 48 to 72 h and processed as previously reported^11^. Slide images were acquired using a Leica Aperio® AT2 digital scanner and captured at 40x magnification with a resolution of 0.25 µm pixel^−1^.

### Fecundity and mRNA expression of adult WCR exposed to dsRNA *vgr* (ds*vgr*) or *bol* (ds*bol*)

The detailed insect rearing methods are described in Supplementary Methods B. Detailed description of fecundity assessment and estimation of net reduction in fecundity (NRF) are described in the Supplementary Methods C and D. Adult WCR insect bioassays were carried out using a diet incorporation methodology by incorporating dsRNA into previously described artificial diet^42,43^. To create the adult artificial diet pellets used in the bioassays, 25 µl of a solubilized solution of dsRNA (ds*vgr*, ds*bol*, ds*gfp*, or ds*gus*) was mixed with 75 µl of WCR artificial diet and placed in the well of a 96-well microtiter plate, for a final concentration of 100 ng µl^−1^ of dsRNA. For control diet, 25 µl of sterile deionized water was incorporated into 75 µl of WCR artificial diet per well. Adult beetles from the same batch of eggs were separated into two groups: young adults (<5 days old) and old adults (>11 days old). The following three treatments were compared 1) sterile deionized water (control); 2) *gfp* dsRNA (ds*gfp*), and 3) *vgr* dsRNA (ds*vgr*). For exposure, individual WCR adult beetles were confined for 24 h in individual wells of 32 cell tray (C-D International, Pitman, NJ) supplemented with a single artificial diet pellet, containing the desired treatment as described above. After 24 h, treated adults were transferred to their respective cages (30 × 30 × 30 cm popup cages with vinyl window; Raising Butterflies LLC, Salt Lake City, UT) to assess fecundity (Supplementary Methods E). Exposure of old adults to 100 ng μl^−1^ dsRNA *bol* (ds*bol*) was described in Supplementary Method F.

### Fecundity of WCR exposed to ds*vgr* and ds*bol* at 3^rd^ instar stage

3^rd^ instar larvae were acclimatized on WCR larval diet^44,45^ for 24 h and exposed to a diet containing sterile deionized water (control) or 50 ng μl^−1^ of *vgr* dsRNA (ds*vgr*), *bol* dsRNA (ds*bol*) or *gus* dsRNA (ds*gus*) (Supplementary Methods G). A total of 18 diet-acclimatized 3^rd^ instar larvae were added to each well of a 6-well costar plate (Corning Incorporated, Corning, NY) (n = 108 larvae per plate). Four replicate plates were prepared per treatment, for a total of 432 3^rd^ instar larvae per treatment. After 24 h exposure to dsRNA, larvae were transferred to a pupation dish (clear plastic container 18.7 cm diameter by 7.6 cm height, (Pioneer Plastics, Dixon, KY) filled with Miracle-Gro Garden soil (Scotts Company, Marysville, OH)) and incubated until adult emergence. Prior to the onset of adult emergence, each pupation dish was placed into a separate cage and both food and water source were added. Emerged beetles were counted, sorted by sex and treatment at the end of the 10-day pre-oviposition period. Beetles were randomly picked from male or female cages of the respective treatment to create three replicate cages for the fecundity study (10 to 22 pairs per cage). Each cage received a new oviposition dish every 5 days for 15 days.

For further assessment of 3^rd^ instar response to different concentrations of ds*bol* or ds*vgr*, a dose-response assay was conducted with four different concentrations (0.1, 1, 10, and 50 ng µl^−1^ of ds*bol* or ds*vgr* in diet). Diet acclimatized 3^rd^ instar larvae (n = 432) were exposed for 24 h to target doses of ds*bol* or ds*vgr*, and were incubated as described above to complete development. Emerged beetles were handled as described above, and at the end of a 10 d pre-oviposition period, four replicate cages containing 10 to 19 pairs were arranged for the fecundity study. Egg collection was performed every 5 days for 15 and 25 days for the ds*vgr* and ds*bol* assays, respectively, and egg hatch was assessed over a period of 8 days.

### Sex-specific effects of ds*bol* exposure at 3^rd^ instar stage via reciprocal crossing

Diet acclimatized 3^rd^ instar larvae were exposed for 24 h to an artificial diet containing 50 ng µl^−1^ of ds*bol* or water control. A total of 3,780 3^rd^ instar larvae were exposed for each treatment. After 24 h, treated larvae were transferred to individual pupation cup (37 ml capacity translucent plastic cup with clear lids; Dart container corporation, Michigan, USA) filled with moist soil and were incubated to complete development. Emerged beetles were sorted, and placed in cages by sex and treatment. For the fecundity study, four reciprocal crossing combinations (*bol* ♂ x *bol* ♀; bol ♂ x H_2_O ♀; H2O ♂ x *bol* ♀; H_2_O ♂ x H_2_O ♀) were arranged in six replicate cages (n = 18 to 24 pairs per cage). The experiment was conducted for 25 days, and egg production and egg hatch rate was assessed following a similar procedure described above.

### Fecundity of WCR feeding on plants expressing *vgr* and *bol* dsRNA transcripts

Eight T1 transgenic maize lines expressing the *vgr* dsRNA transcript (three fragments), and three T1 lines expressing the *bol* dsRNA transcript were produced and characterized, as described in the Supplemental Methods H and Supplementary Table 3. For each transgenic and non-transgenic (NTG) control line, three T1 plants were transplanted into a plastic pot (for a total of 7 to 11 pots per maize line), and pots were maintained in the greenhouse (27 °C, photoperiod of 15: 9 (L: D) h. Each pot was used as a replicate for exposure. At the V2 growth stage, each pot was infested with 200 WCR eggs. After 30 days, plants/pots were monitored daily for beetle emergence. Adult beetles were collected following previously described methods^46^ and were brought to the laboratory every 2 to 3 days, for two to three weeks. Beetles were counted, sexed and kept in cages by treatment. At the end of the pre-oviposition period, females were recounted and randomly picked to create replicate cages (n = 3; 8 to 16 pairs per cage). Experimental cages were maintained as described above for 15 and 25 days, for *vgr* and *bol* transgenic plants, respectively. For the *vgr* treatment, eggs were collected daily or at an interval of 2–4 days for total observation of (n = 9). For the *bol* treatment, egg collection was performed every 5 days.

### Data Analysis

Egg numbers and percent egg hatch data was transformed using log10 or arcsine square-root, respectively to satisfy normality and homogeneous variance assumptions. Statistical analyses were performed using PROC GLM or PROC MIXED model. When a significant difference was detected, pairwise comparison of means was performed following the least square mean (LSMEANS) procedure in SAS Enterprise Guide v6.1 (SAS Institute, 2013).

The daily egg numbers and hatch data was considered to be independent and a one-way or two-way ANOVA was performed using PROC GLM. When treatments were replicated and data was collected for multiple time point, the data were analyzed using PROC MIXED procedure. The cage to cage variability was removed from the overall error, using the cage as a random effect. The treatment effect at each time point was estimated and tested at an alpha level of 5%. Insect and plant expression data were subjected to one-way- analysis of variance using JMP (v12. SAS Institute Inc, Cary, NC) followed by Dunnett’s post-test. For all analysis, the results were considered statistically significant if the *P*–value was <0.05.

### Data availability

The RNAi active target sequences have been deposited in the GenBank of National Center for Biotechnology Information under the accession numbers KY373243 and KY373244.

## Electronic supplementary material

Supplementary information

## References

[1] Gray ME, Sappington TW, Miller NJ, Moeser J, Bohn MO (2009). Adaptation and invasiveness of western corn rootworm: intensifying research on a worsening pest. Annu. Rev. Entomol..

[2] Narva KE, Siegfried BD, Storer NP (2013). Transgenic approaches to western corn rootworm control. Adv. Biochem. Eng. Biotechnol..

[3] Levine E, Oloumi-Sadeghi H (1991). Management of Diabroticite Rootworms in Corn. Annu. Rev. Entomol..

[4] Tabashnik BE, Brevault T, Carriere Y (2013). Insect resistance to Bt crops: lessons from the first billion acres. Nat. Biotech..

[5] Gassmann AJ, Petzold-Maxwell JL, Keweshan RS, Dunbar MW (2011). Field-Evolved Resistance to Bt Maize by Western Corn Rootworm. PLoS One.

[6] Jakka SRK, Shrestha RB, Gassmann AJ (2016). Broad-spectrum resistance to Bacillus thuringiensis toxins by western corn rootworm (*Diabrotica virgifera virgifera*). Sci. Rep..

[7] Katoch, R., Sethi, A., Thakur, N. & Murdock, L. RNAi for Insect Control: Current Perspective and Future Challenges. *Appl. Biochem. Biotech*., 1–27 (2013). 10.1007/s12010-013-0399-4 23904259

[8] Baum JA (2007). Control of coleopteran insect pests through RNA interference. Nat. Biotech..

[9] Ivashuta S (2015). Environmental RNAi in herbivorous insects. RNA.

[10] Bolognesi R (2012). Characterizing the mechanism of action of double-stranded RNA activity against western corn rootworm (*Diabrotica virgifera virgifera* LeConte). PLoS One.

[11] Hu X (2016). Discovery of midgut genes for the RNA interference control of corn rootworm. Sci. Rep..

[12] Li H (2015). Long dsRNA but not siRNA initiates RNAi in western corn rootworm larvae and adults. J. Appl. Entomol..

[13] Ramaseshadri P (2013). Physiological and Cellular Responses Caused by RNAi- Mediated Suppression of Snf7 Orthologue in Western Corn Rootworm (*Diabrotica virgifera virgifera*) Larvae. PLoS One.

[14] Khajuria C (2015). Parental RNA interference of genes involved in embryonic development of the western corn rootworm, *Diabrotica virgifera virgifera* LeConte. Insect Biochem. Mol. Biol..

[15] Sappington TW, Raikhel S (1998). A. Molecular characteristics of insect vitellogenins and vitellogenin receptors. Insect Biochem. Mol. Biol..

[16] Lu K (2015). Molecular characterization and RNA interference analysis of vitellogenin receptor from *Nilaparvata lugens* (Stal). J. Insect Physiol..

[17] Upadhyay, S. K., Singh, H., Dixit, S., Mendu, V. & Verma, P. C. Molecular characterization of vitellogenin and vitellogenin receptor of *Bemisia tabaci*. *PLoS One* **11** (2016). 10.1371/journal.pone.0155306 PMC4861306 27159161

[18] Zhang, W. *et al*. Molecular characterization and function analysis of the vitellogenin receptor from the cotton bollworm, *Helicoverpa armigera* (Hubner) (Lepidoptera, Noctuidae). *PLoS One* **11** (2016). 10.1371/journal.pone.0155785 PMC4871585 27192057

[19] Lin Y (2013). Vitellogenin Receptor Mutation Leads to the Oogenesis Mutant Phenotype “*scanty vitellin*” of the Silkworm, *Bombyx mori*. J. Biol. Chem..

[20] Sekine K, Furusawa T, Hatakeyama M (2015). The *boule* gene is essential for spermatogenesis of haploid insect male. Dev. Biol..

[21] Castrillon DH (1993). Toward a molecular genetic analysis of spermatogenesis in *Drosophila melanogaster*: characterization of male-sterile mutants generated by single P element mutagenesis. Genetics.

[22] Eberhart CG, Maines JZ, Wasserman SA (1996). Meiotic cell cycle requirement for a fly homologue of human *Deleted in Azoospermia*. Nature.

[23] Fu XF (2015). DAZ Family Proteins, Key Players for Germ Cell Development. Int. J. Biol. Sci..

[24] Gramates LS (2017). FlyBase at 25: looking to the future. Nucleic Acids Res..

[25] Dietzl G (2007). A genome-wide transgenic RNAi library for conditional gene inactivation in Drosophila. Nature.

[26] Schonbaum CP, Lee S, Mahowald AP (1995). The Drosophila *yolkless* gene encodes a vitellogenin receptor belonging to the low density lipoprotein receptor superfamily. Proc. Natl. Acad. Sci. USA.

[27] Whyard S (2015). Silencing the buzz: a new approach to population suppression of mosquitoes by feeding larvae double-stranded RNAs. Parasit. Vectors.

[28] Dong, Y. C., Wang, Z. J., Chen, Z. Z., Clarke, A. R. & Niu, C. Y. *Bactrocera dorsalis* male sterilization by targeted RNA interference of spermatogenesis: Empowering sterile insect technique programs. *Sci. Rep*. **6**, doi:10.1038/srep35750 (2016). 10.1038/srep35750 PMC5073305 27767174

[29] Pereira AE, Carneiro NP, Siegfried BD (2016). Comparative susceptibility of southern and western corn rootworm adults and larvae to vATPase-A and Snf7 dsRNAs. J. RNAi and Gene Silencing.

[30] Steiner JK, Tasaki J, Rouhana L (2016). Germline Defects Caused by *Smed-boule* RNA-Interference Reveal That Egg Capsule Deposition Occurs Independently of Fertilization, Ovulation, Mating, or the Presence of Gametes in Planarian Flatworms. PLoS Genet..

[31] Iyer H, Issigonis M, Sharma PP, Extavour CG, Newmark PA (2016). A premeiotic function for *boule* in the planarian *Schmidtea mediterranea*. Proc. Natl. Acad. Sci. USA.

[32] Tung JY (2006). Evolutionary comparison of the reproductive genes, DAZL and BOULE, in primates with and without DAZ. Dev. Genes Evol..

[33] Zhang J (2015). Full crop protection from an insect pest by expression of long double-stranded RNAs in plastids. Science.

[34] Li H (2016). Systemic RNAi in western corn rootworm, *Diabrotica virgifera virgifera*, does not involve transitive pathways. Insect Sci..

[35] Harari, A. R., Sharon, R. & Weintraub, P. G. In *Adv. Insect Control and Resistance Manag*. (eds A. Rami Horowitz & Isaac Ishaaya) 93–119 (Springer International Publishing, 2016).

[36] Leftwich PT, Bolton M, Chapman T (2015). Evolutionary biology and genetic techniques for insect control. Evol. Appl..

[37] Tabashnik BE (2010). Suppressing resistance to Bt cotton with sterile insect releases. Nat. Biotech..

[38] Grabherr MG (2011). Full-length transcriptome assembly from RNA-Seq data without a reference genome. Nat. Biotech..

[39] Apweiler R (2001). The InterPro database, an integrated documentation resource for protein families, domains and functional sites. Nucleic Acids Res..

[40] Ho SN, Hunt HD, Horton RM, Pullen JK, Pease LR (1989). Site-directed mutagenesis by overlap extension using the polymerase chain reaction. Gene.

[41] Cho MJ (2014). Agrobacterium-mediated high-frequency transformation of an elite commercial maize (*Zea mays* L.) inbred line. Plant Cell Rep..

[42] Rangasamy M, Siegfried BD (2012). Validation of RNA interference in western corn rootworm *Diabrotica virgifera virgifera* LeConte (Coleoptera: Chrysomelidae) adults. Pest Manag. Sci..

[43] Nowatzki TM, Zhou X, Meinke LJ, Vaughn T, Siegfried BD (2006). Effect of *Bacillus thuringiensis* cry3Bb1 protein on the feeding behavior and longevity of adult western corn rootworms (Coleoptera: Chrysomelidae). J. Econ. Entomol..

[44] Zhao JZ (2016). mCry3A-Selected Western Corn Rootworm (Coleoptera: Chrysomelidae) Colony Exhibits High Resistance and Has Reduced Binding of mCry3A to Midgut Tissue. J. Econ. Entomol..

[45] Schellenberger U (2016). A selective insecticidal protein from *Pseudomonas* for controlling corn rootworms. Science.

[46] Meihls LN (2008). Increased survival of western corn rootworm on transgenic corn within three generations of on-plant greenhouse selection. Proc. Natl. Acad. Sci. USA.

