# Supplementary material for: Control of Western Corn Rootworm (Diabrotica virgifera virgifera) Reproduction through Plant-Mediated RNA Interference
Source: Sci Rep. 2017 Oct 3;7:12591. doi: 10.1038/s41598-017-12638-3 (PMC5626700; doi:10.1038/s41598-017-12638-3)
Supplement: Supplementary file 1 — Supplementary information [file 41598_2017_12638_MOESM1_ESM.pdf]

Control of Western Corn Rootworm (*Diabrotica virgifera virgifera*) Reproduction through Plant-Mediated RNA Interference

Xiping Niu[1], Adane Kassa[1], Xu Hu[1*], Jonathan Robeson[1], Mollie McMahon[1], Nina M. Richtman[1], Joseph P. Steimel[1], Bliss M. Kernodle[1], Virginia C. Crane[1], Gary Sandahl[1], Julie L. Ritland[1], James K. Presnail[1,2], Albert L. Lu[1], and Gusui Wu[1]

[1]DuPont Pioneer, 7300 NW 62nd Ave., Johnston, IA,

[2]present address: Evogene Ltd., Saint Louis, MO

X.N. and A.K. made an equal contribution

[*]Corresponding Author: DuPont Pioneer, 7300 NW 62nd Ave. Johnston, IA 50131, USA

Telephone: 515-535-2105

E-mail: xu.hu@pioneer.com

Supplementary Information

Supplementary Methods:

A. Extraction of VgR and BOL proteins from dissected reproductive tissues

Multiple protein extraction methods were being tested to optimize efficient extraction of VgR and BOL proteins from WCR ovary and testes tissue. Dissected ovaries were grounded in 1% b-mercaptoethanol, 0.01% Tween-20, 1% proteinase Inhibitor and 1X PBS (137 mM NaCl, 10 mM Phosphate, 2.7 mM KCl, and a pH of 7.4), and a phenol extraction method [1] was used to extract VgR from the ovary. PBST extraction buffer (Phosphate buffered saline with Tween-20; 8mM Na2HPO4, 150mM NaCl, 2mM KH2PO4, 3mM KCl, 0.05% Tween-20, pH 7.4) was used to extract BOL from dissected testis samples. Protease inhibitor (1%) was added in both extraction buffers to improve protein stability.

B. Insect Source and handling

Non-diapausing WCR, (*Diabrotica virgifera virgifera*) was obtained from the U.S. Department of Agricultural Research Service (USDA-ARS) North Central Agricultural Research Laboratory in Brookings, SD [2]. Before and after exposure to treatments, adult beetles were maintained on southern corn rootworm (SCR) adult dry diet (Frontier Ag Science, product no. F9800B), and 2% agar (wt/vol Type A Agarose, Sigma-Aldrich #A6549) was provided as a water source. Adult cages were maintained at 25 ˚C, 65 ± 5% RH and photoperiod of 14:10 (L: D) h and cage maintenance were performed twice a week. Adult cages (White 30 x 30 x 30 cm popup cages with vinyl window) were obtained from Raising Butterflies LLC, Salt Lake City, UT.

Corn rootworm larvae were reared on corn plants until they reached 3rd instar or adult life stages and were subsequently acclimatized on WCR larval diet for 24 h, prior to being used in bioassays. For third instar larvae, artificial diet acclimatization and the exposure assay plates were incubated at 27 °C, 65 ±

5% RH with continuous light. Larval pupation dishes (clear plastic container 18.7cm diameter by 7.6 cm height (Pioneer Plastics, Dixon, KY) filled with Miracle-Gro Garden soil (Scotts Company, Marysville, OH)) were incubated at 25 or 28 ˚C, 65 ± 5% RH with continuous light depending on the time of adult emergence required.

C. Assessment of WCR Fecundity

In the context of the current study, fecundity is defined as the ability of an insect to produce live progeny. Therefore**,** fecundity parameters (number of eggs produced and egg hatch rate) were measured. To determine the number of eggs produced per female, eggs were collected daily or at an interval of 2-5 days using an oviposition dish (10 x 1.5 cm petri dish containing 1% agar, filter paper disc and 8 layers of moist cheesecloth) and were incubated for 12 to 14 days before processing. The duration of egg collection or incubation varied, depending on the nature of the experiment. Eggs were washed out of oviposition dishes and were suspended in a 0.08 % (wt/vol.) water-agar solution for processing. To determine the number of eggs produced per female, aliquots of egg-agar suspensions were dispensed onto Petri dishes containing water-agar and filter paper. The Petri dishes were imaged and the total number of eggs was counted using an egg counter computer program. The program was developed using Microsoft Visual Studio and OpenCV. Our retrospective power analysis using the initial adult exposure assay egg production data, suggests that 20 to 50 adult females per treatment provided 60 and 92% power, respectively. Thus, for subsequent experiments, we kept the number of test adult females > 40 per treatment. In some instances, however, we used about 20 to 30 females per treatment due to the unexpected low female emergence and /or mortality prior experimental cage setup or due to sampling for gene suppression analysis. For all 3rd instar exposure assays, our target was to obtain enough adult beetles for subsequent egg production studies. Therefore, sample size determination considered the variability of WCR adult emergence rate and

the type of experiment under consideration. For most 3rd instar exposure assays, we used 432 larvae per treatment and used 3780 3rd instar larvae per treatment to get enough adult beetles for the reciprocal crossing study.

To determine the percent of eggs hatched (viable eggs), a 0.08% water-agar solution was amended with an antimicrobial agent to discourage fungal contamination. Aliquot samples (n = 1 to 6, depending on the number of eggs obtained for a given day) containing 25µl of egg–agar suspension were dispensed onto a hatch plate (six well costar plate containing moistened blotting paper (8 x 12 cm), Fisher Scientific Prod. No. NC0787294), and the lids were secured with micropore tape. The average number of eggs in each 25µl egg-agar suspension varies from 11 to 44 depending on a number of eggs available in each treatment on a given day. Egg hatch plates were incubated for a total of 5 to 11 days at 25 ˚C, 65 ± 5% RH with continuous light and egg hatch was assessed over a 3 to 8-day period by counting the number of eggs showing larval emergence hole under a stereomicroscope.

D. Estimation of Net Reduction in Fecundity (NRF)

The number of progeny (viable eggs) produced per female was estimated by multiplying the daily egg number by the proportion of daily egg hatch and was used to determine the net reduction in fecundity (NRF) using the following formula: NRF (%) = $[1-(NVE_t / NVE_{wc})] * 100$. Where "$NVE_t$" is a number of viable eggs in the treatment group and "NVE_{wc}" is a number of viable eggs in water (control) group.

E. Fecundity of adult WCR exposed to dsRNA *vgr* (ds*vgr*) and *bol* (ds*bol*)

For ds*vgr* exposure, adult beetles from the same cohort were categorized into two age groups (young and old). The young adult group (<5 days old) consisted of both of males and females (where the females were in their pre-oviposition period). The old adult group (>11 days old) consisted of only females (that were

mated and in their oviposition period). For each treatment, 50 pairs (females and males) from the young adult group and 50 mated females from the old adult group selected. Individual WCR adult beetles were confined for one day in individual wells of 32 cell tray (C-D International, Pitman, NJ) supplemented with a single artificial diet pellet (containing sterile deionized water, 100ng µl^{-1} ds*gfp*, or 100ng µl^{-1} ds*vgr* prepared in 96-well plates). After one day, treated adults were transferred to their respective cages and provided standard SCR dry adult diet and water source until the end of the study period (22 – 25 days). The young adult group in each respective treatment continued mating for additional 7 days until the end of the 10 day preoviposition period. Eggs were collected using oviposition dishes daily for 13 to 14 days starting 1 day or 7 days after exposure, for the old and young female group, respectively. Egg hatch was assessed over a three-day period, with approximately 1287 to 1650 eggs per treatment being used.

For further assessment of the ds*vgr* treatment effect, a dose-response assay was conducted. Females from the older adult group (> 11 days old) were collected from a different batch of eggs and were exposed individually for one day to an artificial diet plug containing one of the following concentrations of ds*vgr*, 0.01, 0.1, 1, 10, or 75 ng µl^{-1}. After exposure, female beetles (n = 40 to 48) were recollected and placed in their respective holding cages for 18 days to assess fecundity. Oviposition dishes were collected daily or at intervals of 2 to 4 days (for total observation of n = 9). For the egg hatching test, 240 to 1140 eggs were used to create 4 to 7 of 25µl aliquot egg-agar suspensions per dose and NRF was estimated as described previously.

F. Fecundity of adult WCR exposed to dsRNA *bol* (ds*bol*)

For dsRNA *bol* (ds*bol*) feeding, untreated 3rd instar WCR larvae were set to emerge individually using a 50 ml tube (Fisher Sceintifc Cat.No. 0553913) filled with moist Miracle-Gro Garden soil. Emerged adult beetles were kept separately by sex and starved for 24 h prior to exposure. Thirty-two pairs (males and

females) were exposed individually to an artificial diet pellet containing one of the three treatments (sterile deionized water (control); 100ng μl^{-1} ds*bol* or 100ng μl^{-1} ds*gus*). Treated beetles were kept separated for an additional six days to allow for possible gene suppression take place, after which time 14 virgin pairs from the respective treatments were combined in a cage for 10 days for fecundity assessment. Eggs were collected in oviposition dishes daily or at 2-days intervals starting 17 days after initial exposure. Egg handling and hatch test were performed following the same protocol described above. Overall a total of 380 to 484 eggs per treatment were counted for the hatch rate.

G. Artificial diets for 3rd Instar WCR bioassays

For the 3rd instar bioassays, an artificial WCR larval diet was used [3,4]. Larval artificial diet was prepared according to manufacturer's guideline for WCR larval diet (Frontier, Newark, DE) with a few adjustments, including the addition of Formalin at 0.1% (v/v), 0.46% KOH (v/v), and triple antibiotic (Sigma-Aldrich, A5955) at 14% (v/v), and other proprietary improvements. To create the artificial diet, a solubilized solution of dsRNA (ds*vgr,* ds*gus,* or ds*bol)* was mixed with WCR larval diet in a ratio of 1:4 (dsRNA solution: diet) to obtain the desired concertation (which ranged from 0.1 to 50 ng μl^{-1}, depending on the nature of the study). To create the control larval artificial diet treatment, sterile deionized water was incorporated into the diet at the same ratio. Subsequently, 2 ml of prepared diet-sample mixtures was dispensed into each well of the 6-well costar plate (Corning Incorporated, Corning, NY).

H. Molecular characterization of transgenic plants

Seeds from transgenic plants were planted and leaf samples from plants at the V2 growth stage were used for qPCR analysis [5] to determine the copy number of the transgene by detecting sequences near LB (NLB) or RB (NRB) of the T-DNA (Supplementary Table 4). Only PCR positive plants were selected for use in

WCR bioassays or for northern blot or QuantiGene analyses [6]. Total RNA was extracted using the mirVana™ miRNA Isolation kit (Life Technologies, Carlsbad, CA) from leaf samples from T1 transgenic maize plants at the V5 growth stage. Ten µg of total RNA was fractionated on a 1.5% denaturing formaldehyde gel. For siRNA northern blot analysis, 20 µg of total RNA was fractionated on a 15% Criterion™ TBE-Urea Gel (Bio-Rad, Hercules, CA). RNA was blotted onto a Hybond-N+ membrane (Amersham, Little Chalfont, United Kingdom). The blots were pre-hybridized in ExpressHyb™ hybridization solution (Clontech, Mountain View, CA) for 1 h and then hybridized in the same solution as the DNA probe overnight at 65° C for dsRNA and 37°C for siRNA, respectively. The autoradiographs of dsRNA northern blots were digitized by ImageQuant LSA (Fujifilm, Tokyo, Japan), or by phosphor screens and digitized by the Typhoon FLA 9500 (GE Healthcare Bio-Sciences Corp., Piscataway, NJ). For siRNA northern blots, P^{32}-labelled target probes were stripped and the blots were re-probed with normalizing miRNA168a biotin labeled oligo probe. Hybridization and detection conditions were followed exactly as the North2South Chemiluminescent Hybridization and Detection Kit (Pierce Biotechnology, Rockford, IL) outlines, hybridization conditions at 55°C. Chemiluminescent signal was detected by Image Quant LSA (Fujifilm, Tokyo, Japan). A custom QuantiGene probe set matching to *vgr* or *bol* target sequences was designed by QuantiGene assay (Affymetrix, Inc., Santa Clara, CA). The ds*vgr* or ds*bol* was used to generate the standard curve to quantifying dsRNA *in planta* [5]. The concentration of WCR *vgr* or *bol* mRNA in purified samples or root tissues was determined with a QuantiGene 2.0 assay following the manufacturer's instructions. The sequence-specific recognition by the QuantiGene probe set has been described in Supplementary Table 6.

Supplementary Figures and Tables

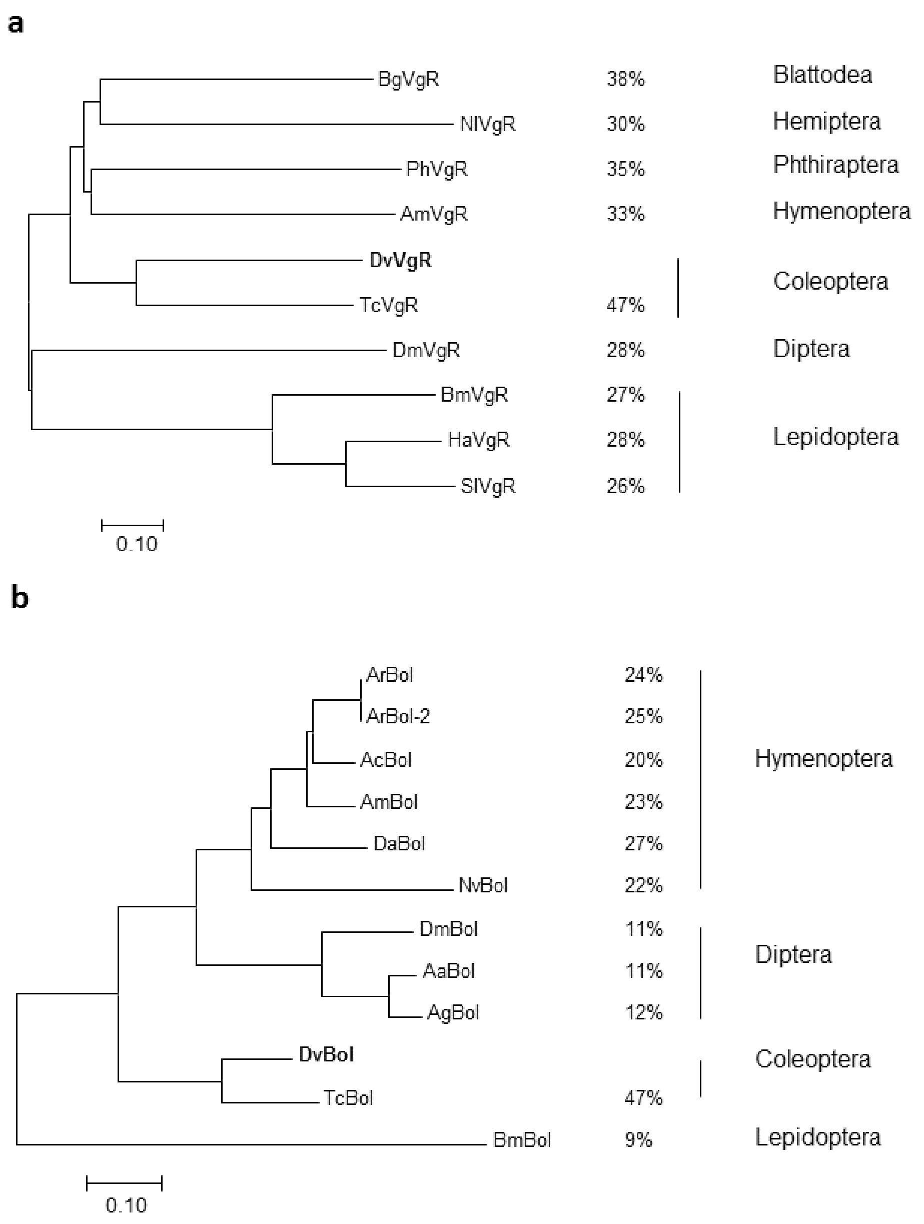

Supplementary Figure 1. Informatics analyses of VgR and BOL protein

Phylogenic tree of insect VgRs (**a**) and BOL (**b**) represents different insect orders, with indications of identity, relative to DvVgR or DvBOL (bold type). Evolutionary analyses were conducted in MEGA7 [7]. The evolutionary history was inferred using the Neighbor-Joining method [8]. The tree is drawn to scale, with branch lengths in the same units as those of the evolutionary distances used to infer the

phylogenetic tree. The evolutionary distances were computed using the Poisson correction method [9] in the units of the number of amino acid substitutions per site. Sequences were retrieved from GenBank database and included the insect VgRs of *Apis mellifera* (AmVgR, XP_001121707), *Blattella germanica* (BgVgR, CAJ19121), *Bombyx mori* (BmVgR, ADK94452), *Drosophila melanogaster* (DmVgR, AAB60217), *Diabrotica virgifera* (DvVgR, KY373243), *Helicoverpa armigera* (HaVgR, AGF33811), *Nilaparvata lugens* (NlVgR, ADE34166), *Pediculus humanus corporis* (PhVgR, XP_002423121), *Spodoptera litura* (SlVgR, ADK94033), and *Tribolium castaneum* (TcVgR, XP_968903). Insect BOL sequences included *Aedes aegypti* (AaBol, AAEL001684), *Atta colombica* (AcBol, KYM84173), *Anopheles gambiae* (AgBol, XP_315505), *Apis mellifera* (AmBol, XP_003250213), *Athalia rosae* (ArBol, AB719980), *Athalia rosae* (ArBol-2, AB719981), *Bombyx mori* (BmBol, ABF51507), *Diachasma alloeum* (DaBol, XP_015116752), *Drosophila melanogaster* (DmBol, AAF50316), *Diabrotica virgifera* (DvBol, KY373244), *Nasonia vitripennis* (NvBol, XP_001599398), and *Tribolium castaneum* (TcBol, EFA05679).

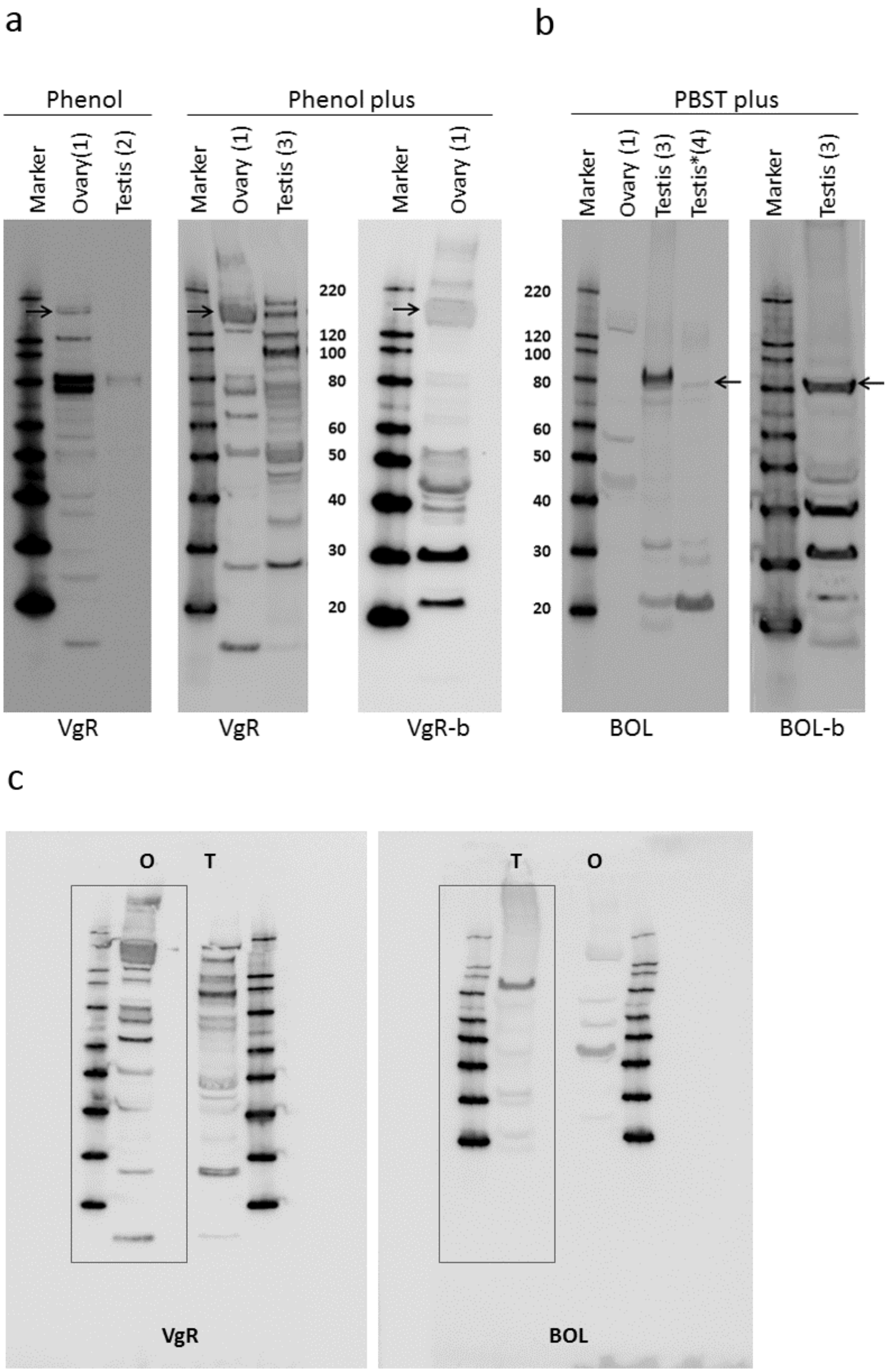

Supplementary Figure 2. ***Diabrotica virgifera virgifera*** **VgR and BOL expression and mRNA expression in different life stages.**

Western analyses of VgR and BOL proteins extracted from dissected reproductive tissues. The presence of VgR and BOL proteins were detected by two separate polyclonal peptide antibodies for targeting different regions of proteins (VgR: DGLGKVTSMDFDPK 690-702a.a, VgR-b: PVREDQKQILKPGQ 1727-1740a.a, BOL: NRRSNDHSSRSSLR 504-517a.a. and BOL-b: VGGKPPAHKSSETI 376-389a.a). Multiple extraction methods were tested to determine optimal extraction of the target proteins. The phenol extraction method was used to extract VgR protein from ovary tissue (**a**) and the PBST method was used to extract the BOL protein from dissected testis samples (**b**). Protease inhibitor was added in both extraction buffers to improve protein stability. Loaded protein samples representing the equivalent number of dissected testis or ovary were indicated in the bracket. The * represents BOL protein that was extracted from testis tissue using the phenol plus method for comparison. The VgR and BOL detectable protein sizes are compared to a Precision Plus Protein Western Standard (Marker) ranging from 20-250 kDa. (**c**) Original pictures of cropped western blots images presented in the results (boxes in Fig. 1c) to detect VgR and BOL from WCR dissected reproductive tissues. Loaded protein samples represent the equivalent of one ovary (O) and 2.5 testes (T).

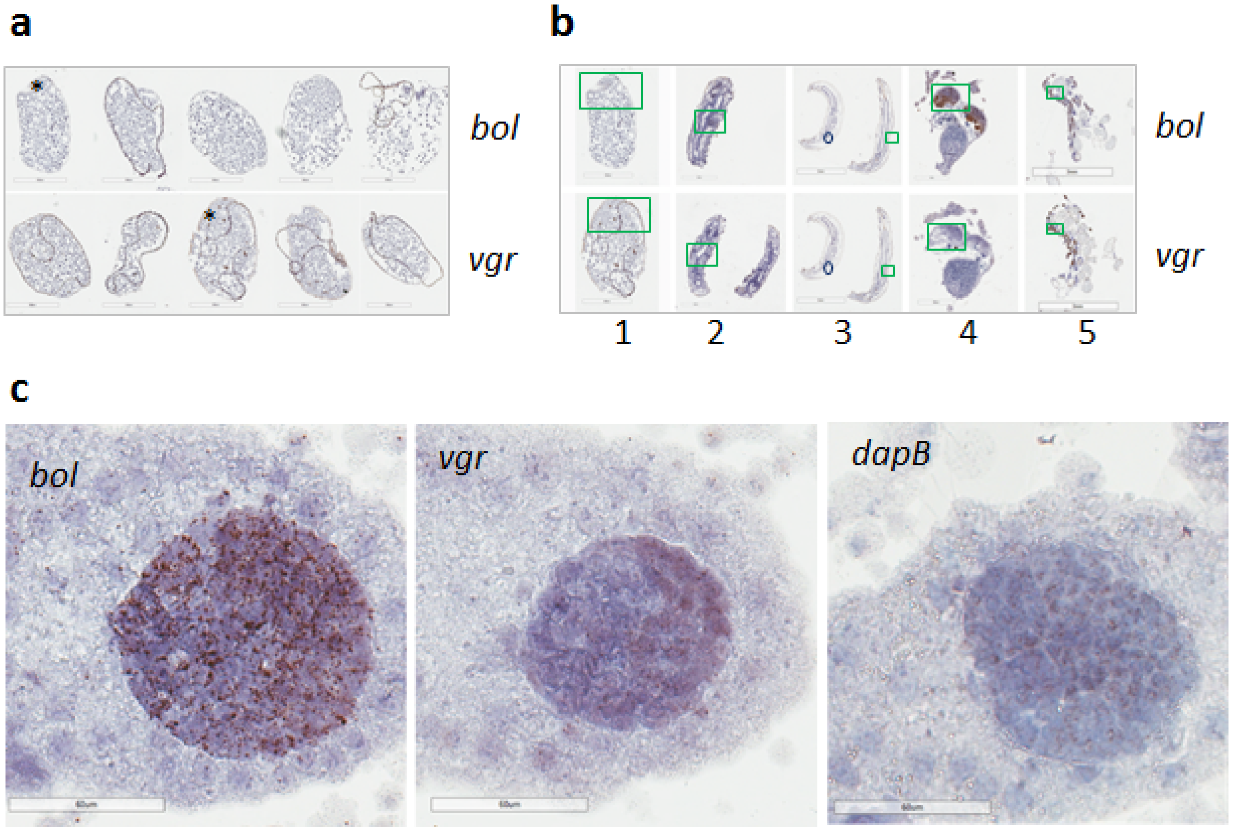

Supplementary Figure 3. Analyses of *vgr* and *bol* mRNA expression during different life stages of *Diabrotica virgifera virgifera* by *in situ* hybridization (ISH).

Multiple insects or dissected reproductive tissues were placed onto slides for *in situ hybridization* and only one or two objects (star, box or circle) were selected for high-resolution images in Fig 2. Adjacent slides of the same 3rd instar or reproductive tissues were used for expression comparison. (**a**) Five egg samples were collected and selected eggs (star) were used for comparison. (**b**) WCR samples were collected at egg (1), neonate (2), 3rd instar of larvae (3), dissected testes (4) and ovaries (5) for *in situ* hybridization. Boxed region corresponds to Fig 2. (**c**) A group of cells may develop into putative gonad (circle in b) in 3rd instar. Images were captured at 40x magnification. Selected samples were hybridized with the *vgr* and *bol* probes and an RNAscope® negative control probe (*Bacillus subtilis* dihydrodipicolinate reductase [*dapB*] gene).

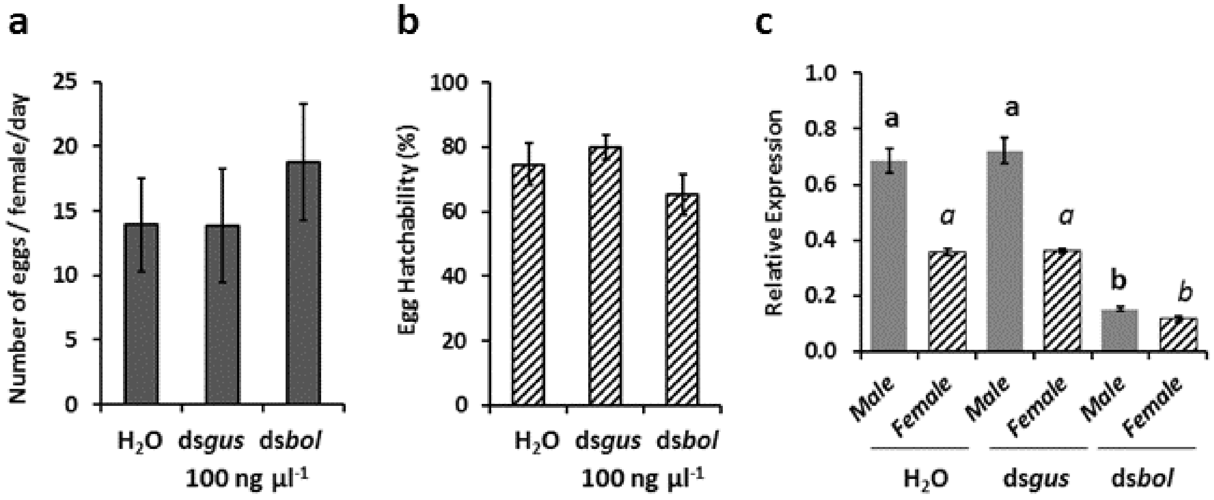

Supplementary Figure 4. Exposure of virgin *Diabrotica virgifera virgifera* adults to ds*bol* did not affect fecundity. Virgin adult beetles were exposed individually to diet containing 100 ng µl $^{-1}$ ds*bol* or sterile water for 24 h. Egg production study was initiated 17 days after exposure and was continued for 10 days. (**a**) Daily egg production per female (mean ± SE) (n = 7). No significant difference between treatments ($P > 0.05$). (**b**) Percent egg hatchability (mean ± SE; n = 5 to 6). No significant difference between treatments ($P > 0.05$). (**c**) Gene suppression analysis of WCR male and female adults 6 days after dsRNA treatment (mean ± SE; n = 6 to 10). Relative expression of *bol* in males and females by qRT-PCR assay is shown for each treatment using *rps10* as a reference and normalized to *bol* expression in water control. Bars followed by the same letters are not significantly different.

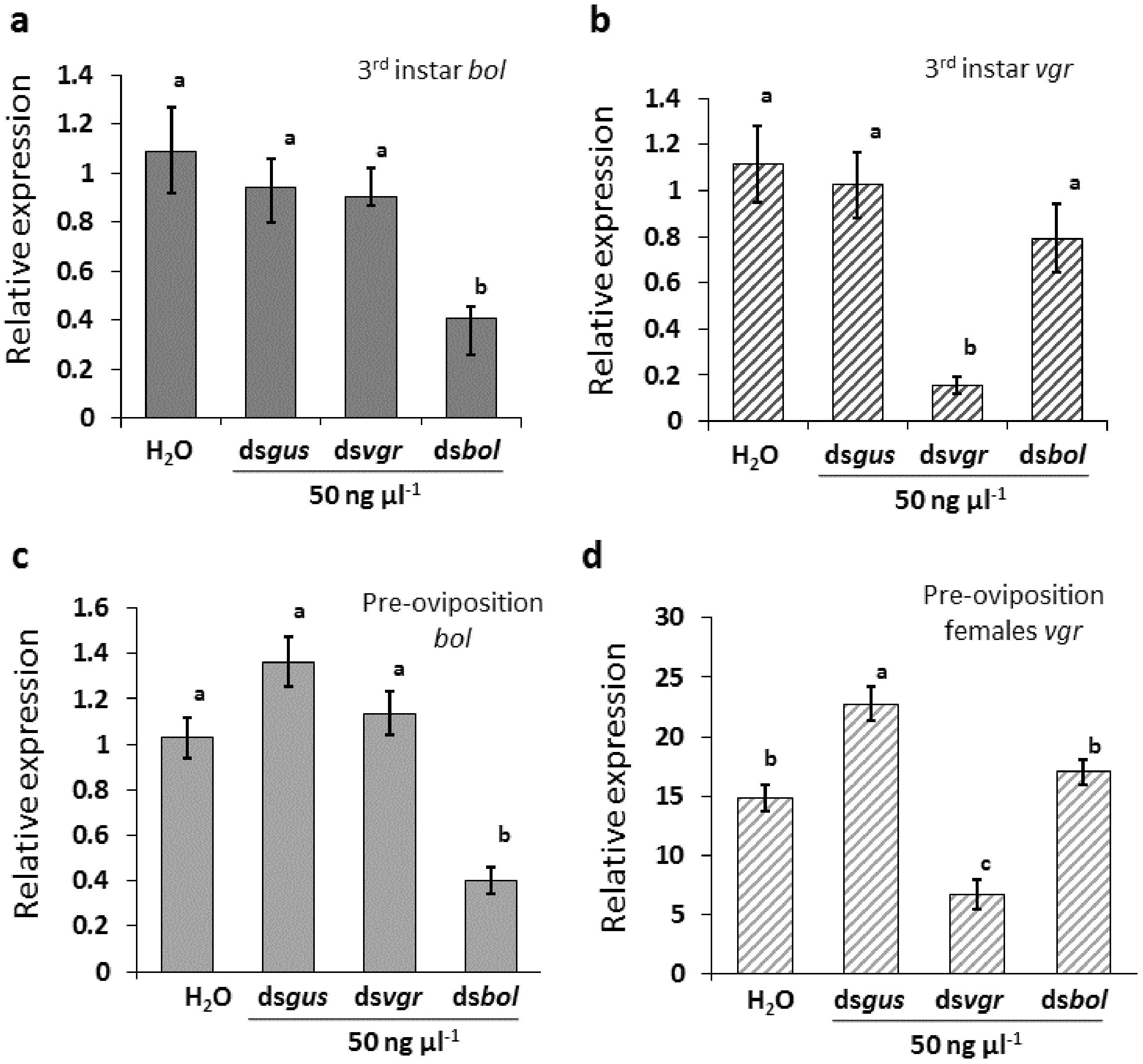

Supplementary Figure 5. Analyses of *bol* and *vgr* mRNA expression after dsRNA exposure.
3rd instar larvae were collected 2 days after exposure to 50 ng µl^{-1} ds*gus*, ds*vgr*, ds*bol* or water (control) for relative expression of *bol* (a) and *vgr* (b) mRNA (mean ±SE). In addition, emerging adult beetles were also measured by qRT-PCR for expression of *bol* (c) and *vgr* (d) mRNA at the pre-oviposition stage. Relative expression is shown for each treatment using *rps10* as a reference and after normalizing to *bol* or *vgr* expression in the water control. Bars followed by the same letters are not significantly different ($P > 0.05$).

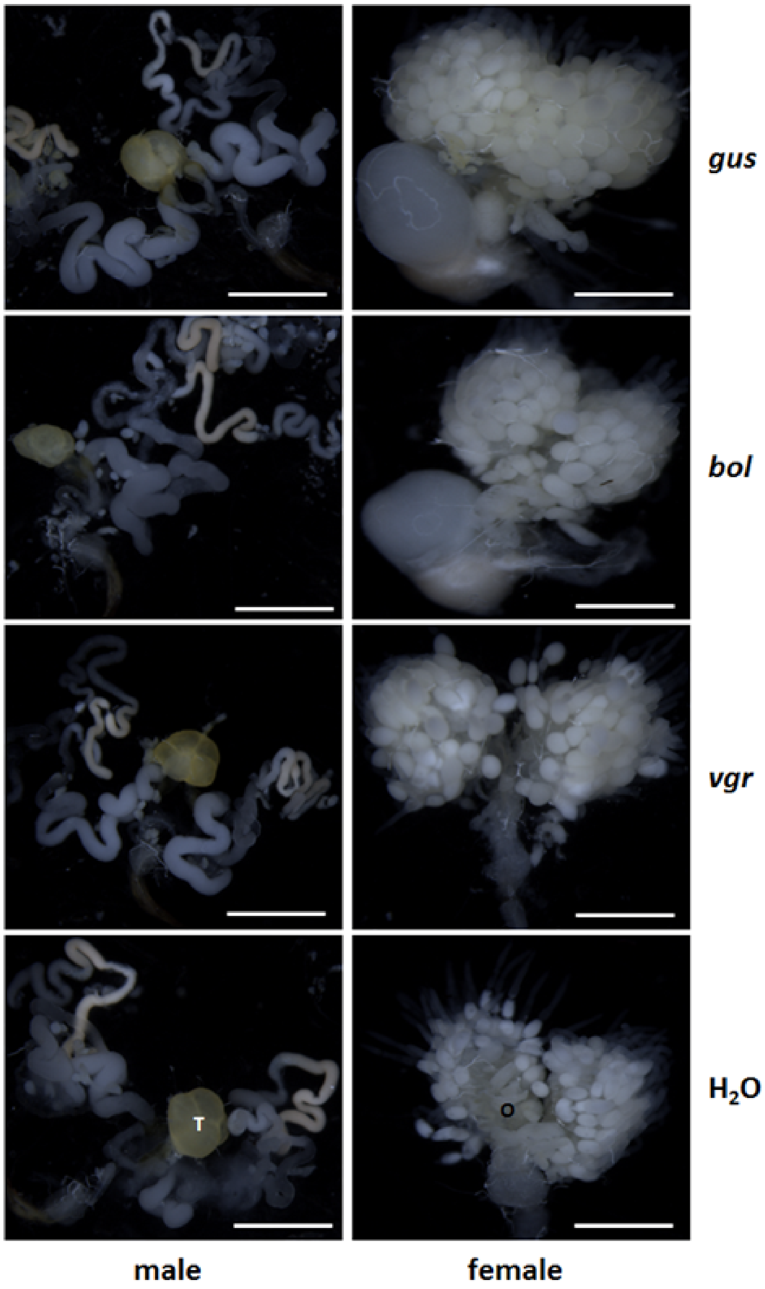

Supplementary Figure 6. Comparison of dissected reproductive tissues from dsRNA-treated *Diabrotica virgifera virgifera* adults. Eight to ten samples (3rd instar exposure of 50 ng μl^{-1} dsRNA) were dissected and examined under dissecting scope. No significant difference was observed among treated samples. Testis (T) and ovary (O) were used for ISH study.

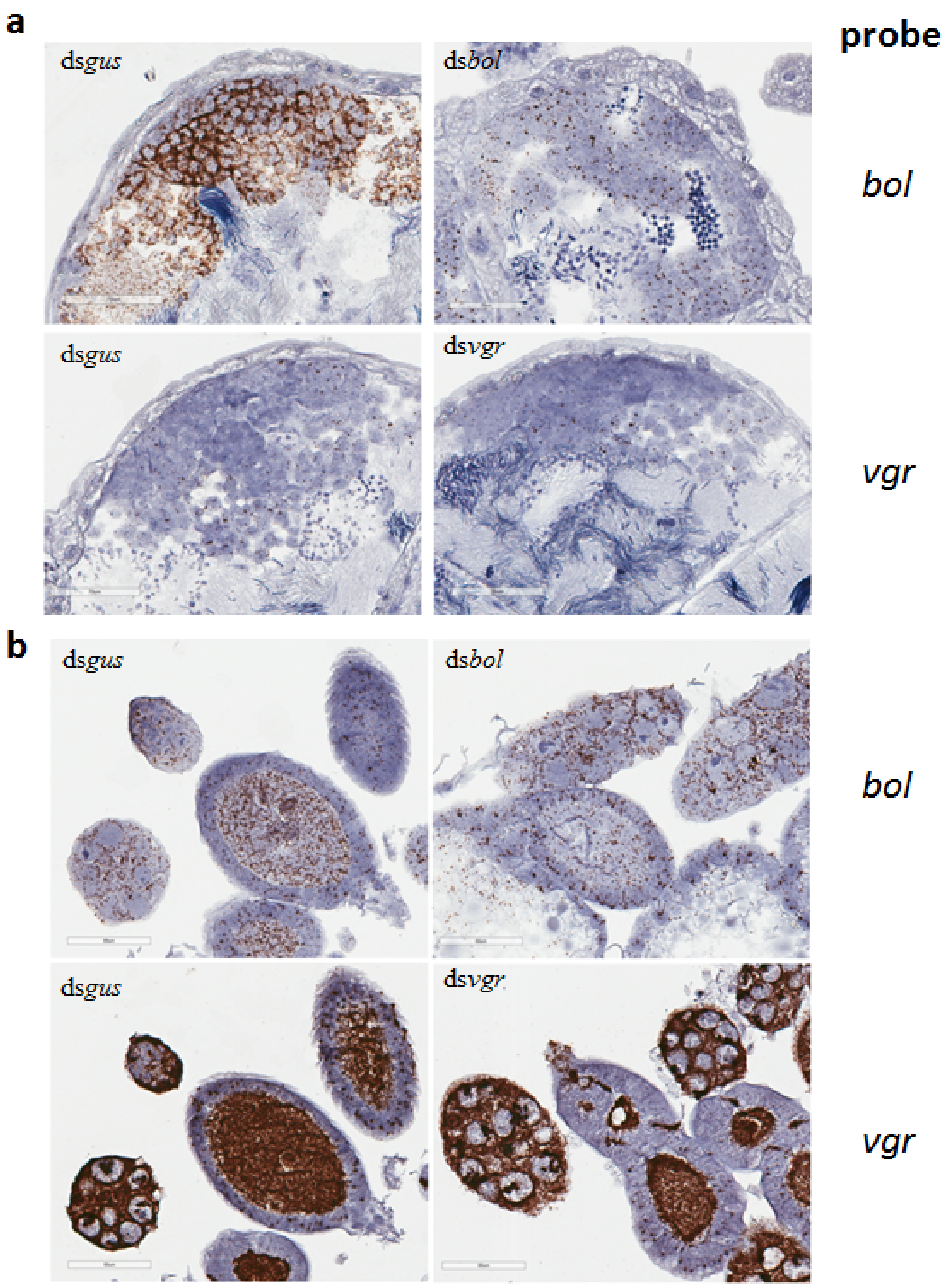

Supplementary Figure 7. Analyses of *bol* and *vgr* mRNA expression in dissected reproductive tissues of *Diabrotica virgifera virgifera*. Testis (**a**) and ovaries (**b**) were dissected from adults treated with *gus*, and *bol* or *vgr* dsRNA at 50 ng μl^{-1} for 48-h.

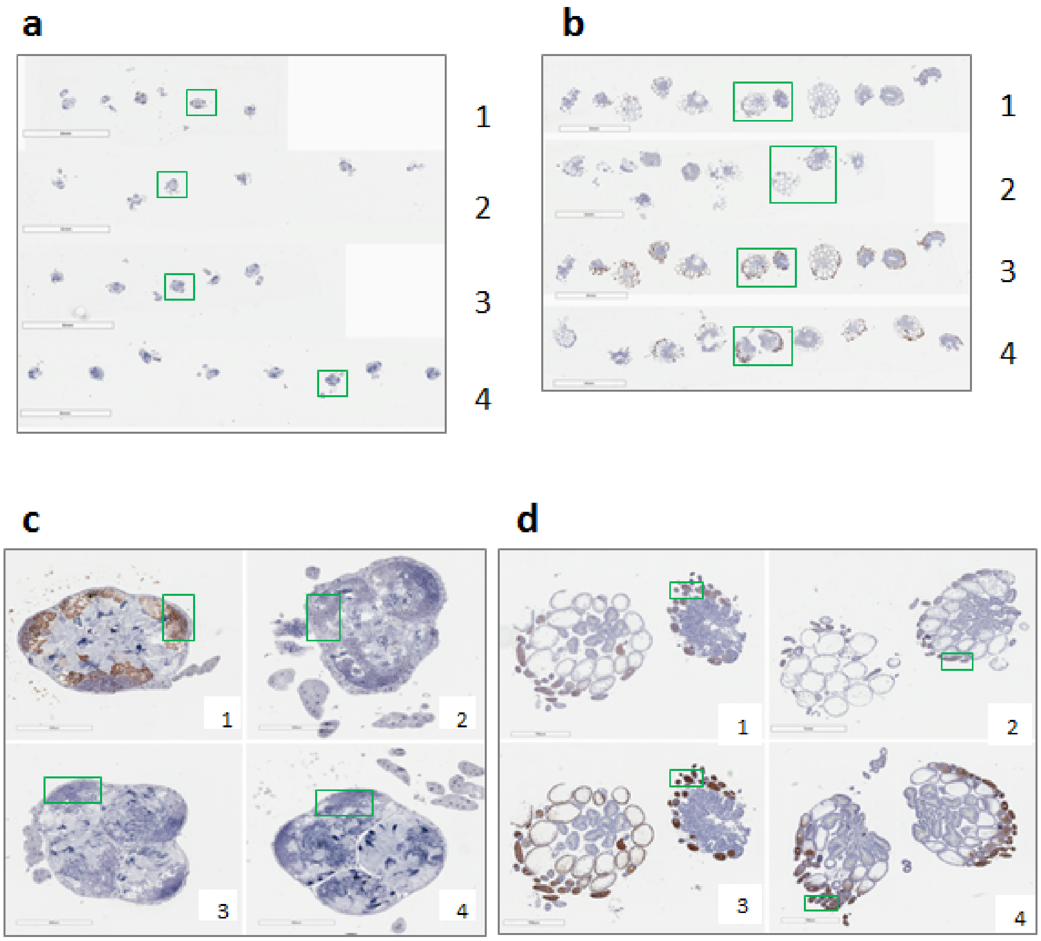

Supplementary Figure 8. Analyses of *dvbol and dvvgr* mRNA expression in the dsRNA-treated samples by ISH. Representative tissue sections (testes (**a**) and ovaries(**b**)) were dissected from ***Diabrotica virgifera virgifera*** adults treated with ds*gus*, and ds*bol* or ds*vgr* at 50 ng μl^{-1} for 48-h. All treated tissue sections were hybridized with the *dvbol* and *dvvgr* probes. At least five samples were examined and only selected objects (green boxes c-testes and d-ovaries) were used for high-resolution image comparison. Knockdown of *bol* RNA is clearly observed in *bol* dsRNA-treated testis (2) in comparison with ds*gus* -treated (1). Boxed images (**c** and **d**) correspond to the images in Supplementary Fig 7. No clear difference of *vgr* expression was detected in *gus* (3) and *vgr* (4) dsRNA-treated testis or ovary. Images (boxed) were captured at 40x magnification with 60 µm scale bars.

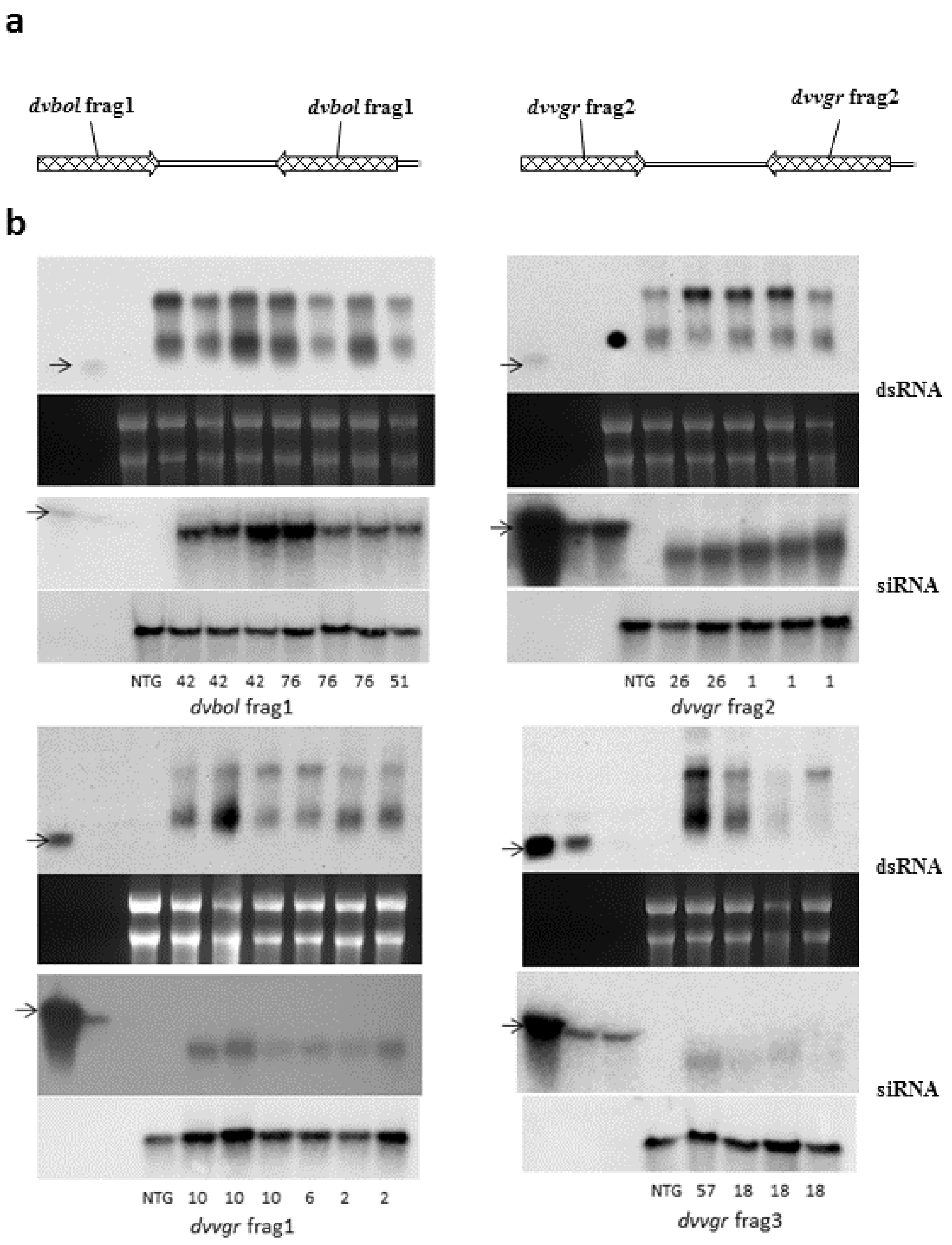

Supplementary Figure 9. Expression analyses of transgenic plants by northern blots. (**a**) Diagram of dsRNA expression cassettes for *dvbol* and d*vvgr* in plant transformation constructs. (**b**) Northern blot analyses of *dvbol* frag1, *dvvgr* frag 1, 2, and 3 constructs. Total RNAs were extracted from root tissues collected from individual transgenic plants representing different lines. Different amounts of dsRNA IVT or oligos (29mer) were added to each blot as positive controls (arrow). For

each construct, the northern image represents dsRNA of the target (top), 28S and 18S ribosomal RNAs (middle), siRNA of target and zm-miR168a (bottom). Visual scores of northern were made and listed in Supplementary Table 3.

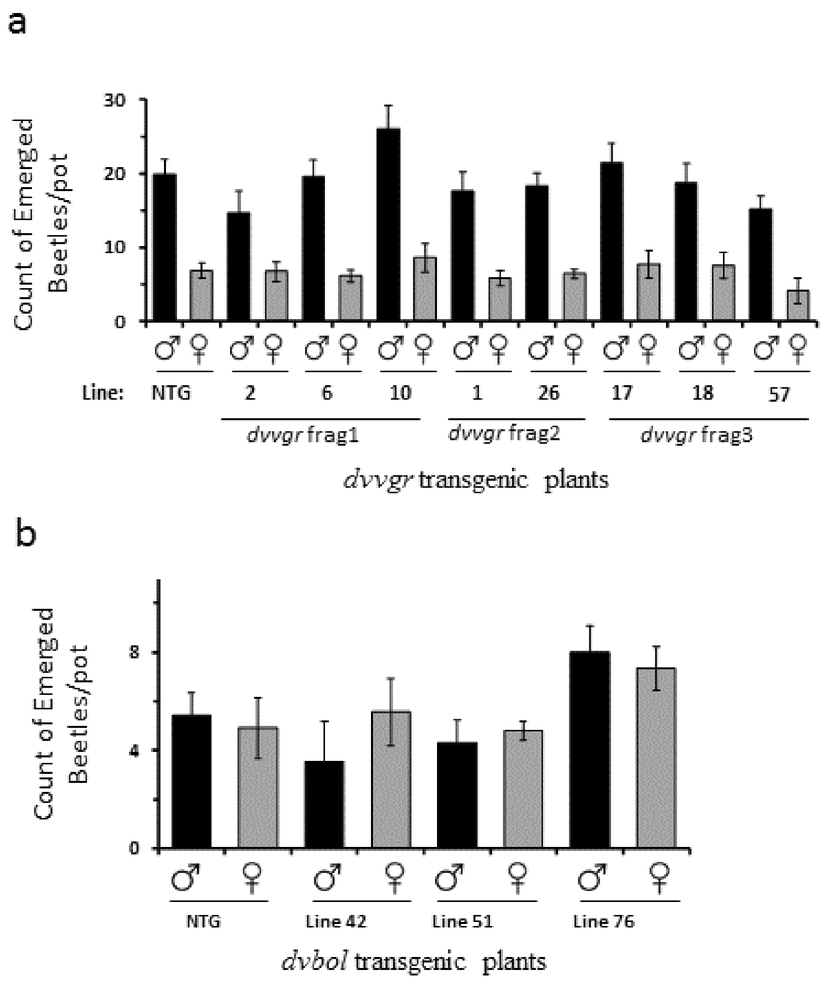

Supplementary Figure 10. Adult emergence (mean ± SE) of *Diabrotica virgifera virgifera* feeding on T1 transgenic roots at larval stages. Adult beetles were collected and sex of each beetle was determined. No statistical difference between control (NTG) and transgenic plants in mean number of adult emergence ($P > 0.05$).

Supplementary Table 1. Dose-dependent effects of ds*vgr* exposure on *Diabrotica virgifera virgifera* fecundity.

ds*vgr* dose (ng µl^{-1})	Total No. eggs laid (18d) [b]	Total No. Eggs / female (18d) [b]	Avg. No. Eggs / female (± SE) (n = 9)		Egg Hatch (%) (± SE) (n = 4 - 7)		Net reduction in Fecundity (%) (± SE) (n = 9)	
75	3236	93	4.0	1.4 **	26.8	13.6 **	78.0	9.5
10	2662	90	5.0	1.1 *	29.7	13.5 **	75.4	11.3
1	2946	109	6.0	1.3	47.1	10.1	68.4	9.0
0.1	4734	165	9.0	1.6	61.3	11.8	46.5	12.2
0.01	6834	227	12.0	1.9	81.9	8.0 a	-24.3	19.9
0	4794	176	10.0	2.1	85.5	3.3 a		
F- value(df)			4.52 (5)		4.13 (5)		10.81 (4)	
P-value			0.0019		0.0061		< 0.0001	

[a] (df): Degrees of freedom.
[b] Statistical analysis was not made
SE*: Standard error of the means*

Adult female beetles (11 days old) were exposed individually to target doses or control for 24 h. After exposure, female beetles were recollected and placed in their respective holding cages for 18-day fecundity study. Least square means pairwise comparison *P*-value from control: > 0.05 (ns, not significant), < 0.05 *, < 0.01 **

Supplementary Table 2. Dose-dependent effects of ds*vgr* exposure at 3rd instar on fecundity of emerged *Diabrotica virgifera virgifera* adult beetles.

Dose (µg /ml)	Total no. eggs laid (15d) (± SE) (n=3)*		No. of eggs / female (15d) (± SE) (n = 3)*		No. of eggs / female/5-day (± SE) (n = 9)		Egg hatch (%) (± SE) (n= 9)		No. Progeny / female (± SE) (n = 3)		Net reduction in Fecundity (%) (± SE) (n = 9)	
50	4165	611.8	216.4	30.9	72.1	21.6	37.6	5.1	83.0	10.5	42.3	10.2 a
10	2815	727.2	298.1	65.0	99.4	41.7	42.7	4.8	129.5	32.9	12.5	14.2 a
1	3373	217.6	327.6	3.1	109.2	21.7	41.2	1.4	135.4	7.5	7.5	5.7 a
0.1	6036	151.2	326.3	9.2	108.8	29.5	51.1	3.1	171.4	8.4	-16.9	14.8 b
0	2697	366.8	282.6	29.7	94.2	27.4	51.8	3.4	146.4	29.1		
Effect test statistics (df)[a]												
Dose					P > 0.05 (4,20)		P > 0.05 (4,20)				P < 0.05 (3,16)	
5-Day					P < 0.05 (2,20)		P < 0.01 (2,20)				P > 0.05 (2,16)	
Dose *5-Day -interaction					P > 0.05 (8,20)		P < 0.01 (8,20)				P > 0.05 (6,16)	

* Statistical analysis was not made

[a] (df): Degrees of freedom and number in parenthesis indicate numerator and denominator degrees of freedom

SE: Standard error of the means

Data showing egg production pattern and egg hatch. Eggs were collected every 5-day. For egg hatch test 103 to 209 eggs/observation was checked for a period of 8 days (total about 1800 to 2008 eggs/dose).

Supplementary Table 3. Molecular characterization of T1 transgenic plants and *Diabrotica virgifera virgifera* fecundity bioassay

T1 expt.	Construct	Line	NRB copy #	NLB copy #	Number of plants	Number of pots	siRNA Northern	dsRNA Northern	RNA pg mg^{-1} fresh weigh ± SE [d]	Total No. Eggs laid ± SE (n= 3) [a]	Total No. eggs / female ± SE (n = 3) [b]	NRF (%) ± SE (n = 15-18) [c]
bol	*bol* frag1	42	1	1	33	11	+++	++++	11.07 (±3.02)	1924 ± 224.5	154 ± 17.2	95.3 ± 2.2 a
		76	1	1	27	9	+++	+++	5.08 (±1.37)	1695 ± 214.5	156 ± 5.5	91.9 ± 3.0 a
		51	2	2	30	10	++	+++	5.45 (±1.13)	2056 ± 93.1	191 ± 17.2	84.1 ± 5.8 a
	control	NTG	0	0	9	3	-	-	0	2052 ± 220.5	243 ± 28.8	
vgr	*vgr* frag1	2	1	1	21	7	++	++	0.61 (±0.29)	3528 ± 169.5	220 ± 10.6	-14.1 ± 13 c
		6	1	1	21	7	++	++	0.63 (±0.35)	3001 ± 149.1	196 ± 12.9	-8 ± 11.3 bc
		10	1	1	21	7	++	+++	0.8 (±0.41)	1643 ± 345	116 ± 8.04	33.5 ± 10.4 a
	vgr frag2	1	1	1	21	7	++	+++	0.89 (±0.56)	2949 ± 187.3	185 ± 11.2	-1 ± 10.4 bc
		26	1	1	21	7	++	++++	1.42 (±0.78)	2334 ± 183.4	150 ± 13.8	15 ± 11.1 ab
	vgr frag3	17	1	1	21	7	n/a	n/a	0.8 (±0.44)	3185 ± 107.5	199 ± 6.7	3.3 ± 10.6 bc
		18	1	1	21	7	+	++	1.12 (±0.49)	2420 ± 321.7	152 ± 19.9	20 ± 9.6 ab
		57	1	1	21	7	++	+++	1.11 (±0.38)	3496 ± 225.9	219 ± 14.1	-9.1 ± 9.6 bc
	control	NTG	0	0	9	3	-	-	0	3138 ± 225.6	202 ± 13.9	

[a,b] n=stands for number of replicate cages per treatment and egg production was assessed for 15 and 25 days for *vgr* and *bol*, respectively. No statistical analysis was made.

[c] Net reduction in fecundity (NRF); number of observation was 15 and 18 for *bol* and *vgr*, respectively. Within the same gene means followed by the same letter are different (least square means).

[d] average of ten T1 plants (root tissue) measured by Quantigene (Supplementary Method and Supplementary Table 7)

Transgenic plants were planted as described in Method. Prior to WCR infestation, leaf and root samples were harvested at the V2 stage for copy number and target expression analyses, respectively (Supplementary Method). Plant samples for northern analyses were planted in a separate experiment. Leaf samples were collected at V2 for analysis of copy number and root tissues were harvested at V5 stage for northern analyses. Northern scores (siRNA and dsRNA) were based on visual estimation of band intensity of target RNA and controls in Supplementary Fig 9.

Supplementary Table 4. List of position and length of *dvvgr*, *dvbol, gfp,* and *gus* fragments relative to their open reading frame (ORF)

Name	Start	End	Length bp	GenBank Accession #
dvvgr ORF	1	5313	5313	KY373243
dvvgr frag1	2	237	236	
dvvgr frag2	21	175	155	
dvvgr frag3	1402	1651	250	
dvbol ORF	1	2085	2085	KY373244
dvbol frag1	160	314	155	
*gfp*ORF	1	717	717	AY233272.1
gfp	258	412	155	
gus ORF	1	1812	1812	S69414.1
gus	1	155	155	

Supplementary Table 5. Primer and oligo sequences for IVT production

Fragment	Primer or oligo designation	Sequence
PCR method		
dvvgr frag1	Forward	TGTTAATTTTGTTCTTTGTGCTTTCAACGTTATTAAATGC
	Reverse	CAGGAATGCTTCGCTTATACATCTTCCATTTCGACAATTG
dvvgr frag2	Forward	CTTTCAACGTTATTAAATGCAGTCGTTTCAACGAATAAT
	Reverse	ATCACAATCCAGTTCATCCGATCCATCTCCACAATTGTC
dvvgr frag3	Forward	TCACCAATAATATACACTGGCCCAATGGTTTGGCTCTAG
	Reverse	TATGATGATTTTTACCATTAAACTTATCACAAGTTTGAAT
Overlap extension PCR method		
dvvgr frag2	5' external forward	GCTTTCAACGTTATTAAATGCAGTCGTTTCAACGAATAAT
	internal reverse	ACATTTTGAATTTGTGCATCTAAACTGATGTGGAGGGCAATCATTATTATTCGTTGAAAC
	internal forward	ACAAATTCAAAATGTATAGACTTTCAACAAAGATGTGATGGTTCCGACAATTGTGGAGAT
	3' external reverse	GATCACAATCCAGTTCATCCGATCCATCTCCACAATTGTC
dvbol frag1	5' external forward	GCTCCAAAGTATGGTACCCTAGTGCCTAACAGAATATTCG
	internal reverse	GTTGCATCAATTCTCCTTCTGTTGTGTTAGCTGATATTCCTCCCACGAATATTCTGTTAG
	internal forward	GAGAATTGATGCAACTTTTTAGCAACTATGGTACTGTTAAAGCTGCTAAAATTATACAGG
	3' external reverse	TATCCTTTTGACACACCAGCCCTGTCCTGTATAATTTTAG
gfp	5' external forward	CGCCATGCCTGAGGGCTACATCCAGGAGCGCACCATCTTC
	internal reverse	GAACTTCACCTCGGCGCGCGACTTGTAGTTGCCGTCATCCTCGAAGAAGATGGTGCGCTC
	internal forward	GCCGAGGTGAAGTTCGAGGGCGATACCCTGGTGAATCGCATCGAGCTGACCGGCACCGAT
	3' external reverse	CCAGGATGTTGCCATCCTCCTTGAAATCGGTGCCGGTCAG
gus	5' external forward	ATGTTACGTCCTGTAGAAACCCCAACCCGTGAAATCAAAA
	internal reverse	CCACAGTTTTCGCGATCCAGACTGAATGCCCACAGGCCGTCGAGTTTTTTGATTTCACGG
	internal forward	ATCGCGAAAACTGTGGAATTGATCAGCGTTGGTGGGAAAGCGCGTTACAAGAAAGCCGGG
	3' external reverse	TTAAAACTGCCTGGCACAGCAATTGCCCGGCTTTCTTGTA

Supplementary Table 6. Summary of probe or primer sequences for molecular analyses

Designation	Annealing or hybridization °C	Sequence or source
**qRT-PCR**		**primer designs for WCR expression study**
dvvgr F	60	AACTCTCGAAACTGATGGCC
dvvgr R		CAAACGATCTTGTTACTGCATCC
dvvgr P (FAM)		AGATGGTCATTGGAAGTGTGCCGA
dvbol F	60	TTGAAAGTGAACAAATAACTAATGCTTAC
dvbol R		AAGATTGGTAATGGGAGTACTGG
dvbol P (FAM)		AGGGGTGTTAACAATAGAGCTGCTTGAA
dvrps10 F	60	CTAACTCTGGCATCGAATACCTC
dvrps10 R		TGGGCGTTTCAAGGTAGATG
dvrps10 P (TET)		TTCTCCAGGTAAGTGTAAGAATGTGCGG
**qPCR**		**primer designs for transgenic copy number**
NLB F	60	TGATTCCGATGACTTCGTAGGTT
NLB R		GCTAATCGTAAGTGACGCTTGGA
NLB P		TAGCTCAAGCCGCTCG
NRB F	60	CATGAAGCGCTCACGGTTACTAT
NRB R		TCGTACGCTACTGCCACCAA
NRB P		ACGGTTAGCTTCACGACT
***In situ* hybridization**		**RNA probes**
dapB probe	40	SKU: 310043; RNAscope® Probe as negative control probe
dvbol RNA probe	40	DvBOL (KY373244) from 100 to 1142nt of orf
dvvgr RNA probe	40	DvVgR (KY373243) from 2 to 1119nt of orf
**Northern analysis**		**probe sequences or source**
zmactin	65	Accession #: EU952376
dvvgr frag1 and 2	65	DvVgR (KY373243) from 2 to 237nt of orf
dvvgr frag3	65	DvVgR (KY373243) from 1402 to 1651nt of orf
dvbol frag1	65	DvBOL (KY373244) from 160 to 429nt of orf
dvvgr f1&2 29 nt control	37	TGTTAATTTTGTTCTTTGTGCTTTCAACG
dvvgr f3 29 nt control	37	GTCACCAATAATATACACTGGCCCAATGG
dvbol 29 nt control	37	GCTCCAAAGTATGGTACCCTAGTGCCTAA
**QuantiGene**		**RNA probe set**
dvbol frag1	54	CTCCAAAGTATGGTACCCTAGTGC<br>CTAACAGAATATTCGTGGGAGGAATATCAGCTAACACAACAGAAGGAG<br>AATTGATGCAACTTTTTAGCAACT<br>ATGGTACTGTTAAAGCTGCTAAAATTATACAGGACAGGGCTGGTGTG
dvvgr frag1-2	54	GCTTTCAACGTTATTAAATGCAGTC<br>CAACGAATAATAATGATTGCCCTC<br>CATCAGTTTAGATGCACAAATTCAA<br>AATGTATAGACTTTCAACAAAGATGTGATGGTTCCGACAATTGTGGAG<br>ATGGATCGGATGAACTGGATTG
dvvgr frag3	54	ACCAATAATATACACTGGCCCAATG<br>GTTTGGCTCTAGATCAACCTAACTCTAGACTTTATTGGACTGATGCTAAAAAGA<br>TGACACTAGAAAGTATTAATTTAGACGGA<br>GATCAAAGGATCGTTTTGGAAGGAATAGTGAAACATCCATATGCCATC<br>TGTTTGAAAACAAACTTTATTGGTCAGATTGGGACTCCCATACCATTCAAA<br>GTGATAAGTTTAATGGTAAAAATCATCA

F=forward primer; R=reverse primer; P=probe

References

1 Hurkman, W. J. & Tanaka, C. K. Solubilization of plant membrane proteins for analysis by two-dimensional gel electrophoresis. *Plant Physiol.* **81**, 802-806 (1986).

2 Branson, T. F. The selection of a non-diapause strain of *Diabrotica virgifera* (Coleoptera: Chrysomelidae). *Entomol. Exp. Appl.* **19**, 148-154, doi:10.1111/j.1570-7458.1976.tb02591.x (1976).

3 Schellenberger, U. *et al.* A selective insecticidal protein from *Pseudomonas* for controlling corn rootworms. *Science* **354**, 634-637, doi:10.1126/science.aaf6056 (2016).

4 Zhao, J. Z. *et al.* mCry3A-Selected Western Corn Rootworm (Coleoptera: Chrysomelidae) Colony Exhibits High Resistance and Has Reduced Binding of mCry3A to Midgut Tissue. *J. Econ. Entomol.*, doi:10.1093/jee/tow049 (2016).

5 Zhi, L. *et al.* Effect of Agrobacterium strain and plasmid copy number on transformation frequency, event quality and usable event quality in an elite maize cultivar. *Plant Cell Rep.* **34**, 745-754, doi:10.1007/s00299-014-1734-0 (2015).

6 Armstrong, T. A. *et al.* Quantification of transgene-derived double-stranded RNA in plants using the QuantiGene nucleic acid detection platform. *J. Agric. Food Chem.* **61**, 12557-12564 (2013).

7 Kumar, S., Stecher, G. & Tamura, K. MEGA7: Molecular Evolutionary Genetics Analysis Version 7.0 for Bigger Datasets. *Mol. Biol. Evol.* **33**, 1870-1874, doi:10.1093/molbev/msw054 (2016).

8 Saitou, N. & Nei, M. The neighbor-joining method: a new method for reconstructing phylogenetic trees. *Mol. Biol. Evol.* **4**, 406-425 (1987).

9 Zuckerkandl, E. & Pauling, L. in *Evolving Genes and Proteins* (eds V. Bryson & H.J. Vogel) 97-166 (Academic Press, 1965).
